# A small molecule ApoE4-targeted therapeutic candidate that normalizes sirtuin 1 levels and improves cognition in an Alzheimer’s disease mouse model

**DOI:** 10.1038/s41598-018-35687-8

**Published:** 2018-12-04

**Authors:** Jesus Campagna, Patricia Spilman, Barbara Jagodzinska, Dongsheng Bai, Asa Hatami, Chunni Zhu, Tina Bilousova, Michael Jun, Chris Jean Elias, Johnny Pham, Gregory Cole, Mary Jo LaDu, Michael E. Jung, Dale E. Bredesen, Varghese John

**Affiliations:** 1 The Drug Discovery Lab, Mary S. Easton Center for Alzheimer’s Disease Research, Department of Neurology, David Geffen School of Medicine, University of California, Los Angeles, CA 90095 USA; 2 Department of Chemistry and Biochemistry, University of California, Los Angeles, CA 90095 USA; 3 Mary S. Easton Center for Alzheimer’s Disease Research, Department of Neurology, David Geffen School of Medicine, University of California, Los Angeles, CA 90095 USA; 4 Veterans Affairs Greater Los Angeles Healthcare System, Los Angeles, CA USA; 5 Department of Anatomy and Cell Biology, University of Illinois at Chicago, Chicago, Illinois 60612 USA; 6 Department of Molecular and Medical Pharmacology, David Geffen School of Medicine, University of California, Los Angeles, CA 90095 USA

## Abstract

We describe here the results from the testing of a small molecule first-in-class apolipoprotein E4 (ApoE4)-targeted sirtuin1 (SirT1) enhancer, A03, that increases the levels of the neuroprotective enzyme SirT1 while not affecting levels of neurotoxic sirtuin 2 (SirT2) *in vitro* in ApoE4-transfected cells. A03 was identified by high-throughput screening (HTS) and found to be orally bioavailable and brain penetrant. *In vivo*, A03 treatment increased SirT1 levels in the hippocampus of 5XFAD-ApoE4 (E4FAD) Alzheimer’s disease (AD) model mice and elicited cognitive improvement while inducing no observed toxicity. We were able to resolve the enantiomers of A03 and show using *in vitro* models that the L-enantiomer was more potent than the corresponding D-enantiomer in increasing SirT1 levels. ApoE4 expression has been shown to decrease the level of the NAD-dependent deacetylase and major longevity determinant SirT1 in brain tissue and serum of AD patients as compared to normal controls. A deficiency in SirT1 level has been recently implicated in increased tau acetylation, a dominant post-translational modification and key pathological event in AD and tauopathies. Therefore, as a novel approach to therapeutic development for AD, we targeted identification of compounds that enhance and normalize brain SirT1 levels.

## Introduction

Alzheimer’s disease (AD) is a progressive neurodegenerative disorder characterized by the presence of senile plaques composed mainly of amyloid-β (Aβ)^1^ peptide and the development of neurofibrillary tangles^2^ in brain tissue. AD patients suffer from deficits in cognition, learning and memory, and impaired cholinergic neurotransmission^3^. Three of the four currently available FDA-approved treatments for AD - donepezil, galantamine, and rivastigmine - provide a modest delay in the cognitive decline of AD patients. These compounds act by enhancing the activity of the neurotransmitter acetylcholine. The fourth FDA-approved treatment, memantine, is a partial antagonist of the N-methyl D-aspartyl (NMDA) receptor. Despite some clinical success of these therapeutic agents, the beneficial effects are limited and last for up to 36 months^4^. No disease-modifying drugs have been approved for clinical use that specifically target the cellular mechanisms of AD, namely the generation of neurotoxic Aβ, tangle-related hyperphosphorylation or acetylation of tau, or other related changes that precipitate onset and contribute to the progression of the disease.

**Figure 1:**
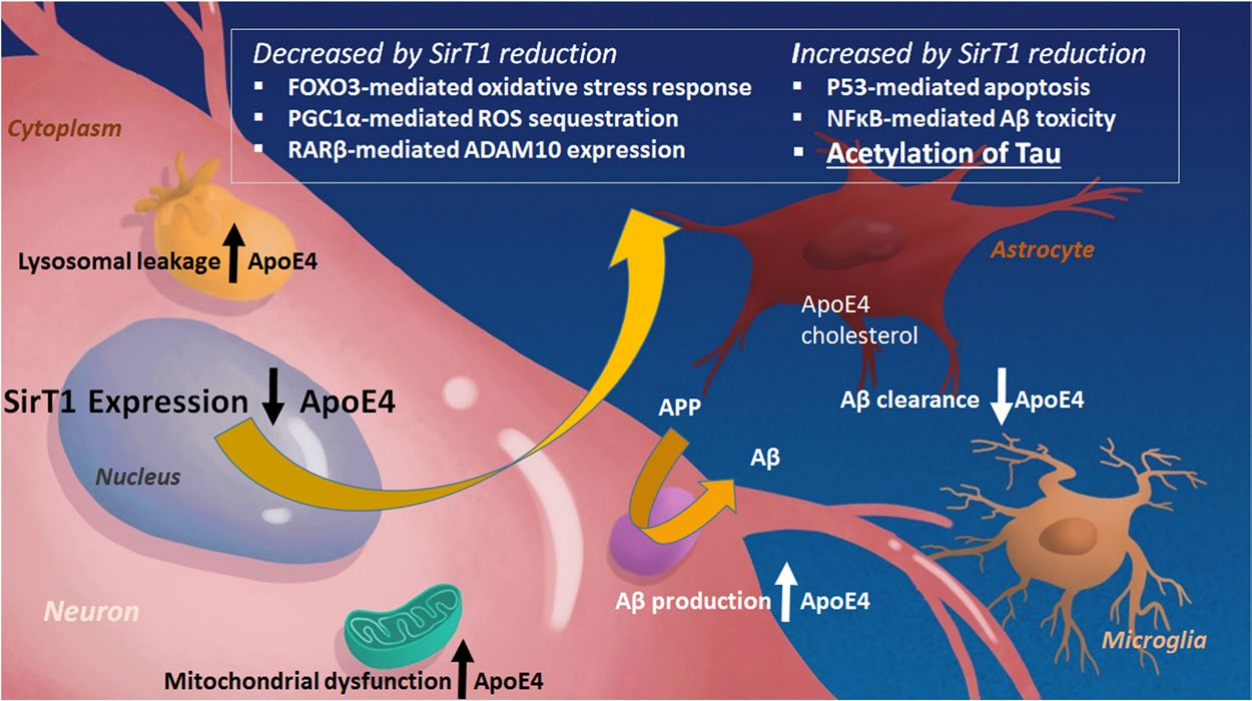
ApoE4 and SirT1 pathways. ApoE4 exacerbates AD-related pathology by increasing β pathway processing of full-length amyloid precursor protein (APP) and therefore Aβ production; and by decreasing astrocyte- and microglia-mediated Aβ clearance. ApoE4 expression is also associated with mitochondrial dysfunction and lysosomal leakage in AD. A reduction of SirT1 as a result of ApoE4 expression may be a major contributor to the deleterious effects of ApoE4 because this can lead to a decrease in the FOXO3-mediated antioxidant response, PGC1α-mediated radical oxygen species (ROS) sequestration, and ADAM10 expression. Decreases in SirT1 also increase p53-mediated apoptosis, NFκB-mediated Aβ toxicity, and acetylation of tau; all of which exacerbate AD pathology.

The major known genetic risk factor for sporadic AD is expression of the epsilon-4 (ε4) allele of apolipoprotein E (ApoE4, E4), which is present in about two-thirds of AD patients^5^. The pathogenesis of AD links the expression of ApoE4 to amyloid precursor protein (APP) processing and amyloid-beta (Aβ) peptide accumulation by a reduction in (Aβ)/amyloid clearance, increased (Aβ) production, and specifically increased toxic Aβ oligomer formation and acceleration during the early seeding stage of (Aβ) aggregation^6^. AD-related neurodegeneration is further exacerbated by ApoE4 through phospholipid dysregulation^7^, mitochondrial dysfunction, and lysosomal leakage (as shown in Fig. 1) to name just a few of the myriad of pro-AD ApoE4 effects^8^. Many researchers have contributed to revealing the complex role of ApoE4 expression in AD and additional mechanisms are still being elucidated^9^.

Our previous studies show that ApoE4 - but not ApoE3 - significantly reduced sirtuin 1 (SirT1) expression in ApoE-transfected cells and resulted in a marked reduction of the ratio of neuroprotective SirT1 to neurotoxic SirT2, triggered tau and APP phosphorylation, and induced programmed cell death^10^. While there are no published studies on the relationship of ApoE4 expression in humans and SirT1 levels, perhaps due to the design requirements for such studies, it was also reported in Theendakara *et al*.^10^ that SirT1 is significantly lower in post-mortem temporoparietal regions of AD brain as compared to cognitively normal controls. This confirmed an earlier report by Julien *et al*.^11^, wherein they revealed there was a significant reduction in both SirT1 mRNA and protein in the parietal cortex of AD patients, and that this reduction correlated with Aβ and tau pathology, and the duration of AD symptoms. Correlations between ApoE4 expression, SirT1 levels, and AD-related mechanisms have been seen in an animal model, including detection of a decrease in SirT1 mRNA in the frontal cortex of ApoE4 mice^12^ and altered expression of proteins that increase the activity of the β-secretase responsible for the first step in Aβ generation, BACE1^13^. Furthermore, in Theendakara *et al*.^14^, ApoE4 was shown to modulate transcription of genes associated with trophic support, programmed cell death, synaptic function, and insulin resistance; all of which may increase the risk for AD. As part of the latter study, a small molecule compound library was screened in human A172 glioblastoma cells transiently transfected with ApoE4 (A172-E4) using SirT1 mRNA levels and enzyme activity as readouts, and several hits that increased the transcription of SirT1 were identified including A03, a known selective serotonin re-uptake inhibitor (SSRI) called alaproclate^14^.

Recent reports indicate that ApoE4-mediated reduction in SirT1 levels has particular importance in AD and point to a critical role for SirT1 in deacetylation of the microtubule-stabilizing protein tau^15^. Increased tau acetylation due to reduced SirT1 levels likely leads to microtubule instability and spread of tau pathology. Interestingly, restoration of SirT1 levels can attenuate propagation of tau pathology, as shown by Min *et al*.^15^. Thus, alterations in brain levels of SirT1 should be considered a key target for AD drug discovery^16^.

Others have identified SirT1 enhancers such as the polyphenolic compounds resveratrol^17^, quercetin, and fisetin^18^ and synthetic compounds SRT1720 and SRT1460^19,20^. While these compounds have shown some CNS effects^21,22^, none has a pharmacokinetic (PK) profile that suggests good oral brain bioavailability^22–26^ particularly when compared to A03. Resveratrol was tested in the clinic^27^ and produced mixed results that are still under analysis; resveratrol will still face hurdles in development due to its poor PK profile, instability and light sensitivity. We wanted to identify an orally available small molecule SirT1 enhancer that would be selective for enhancement of SirT1 over other sirtuins, be effective in the presence of ApoE4, has good brain permeability and an excellent safety profile.

We present here for the first time our findings that support A03 as a potential ApoE4-targeted therapeutic candidate. We describe the setup and optimization of a high-throughput screen (HTS) utilizing murine neuroblastoma N2a cells stably transfected with ApoE4 (N2a-E4) and a customized SirT1 AlphaLISA assay to screen the NIH Clinical Collection module from the UCLA compound library (http://www.mssr.ucla.edu/libraries.html), resulting in confirmation of A03’s SirT1-enhancing effects while not significantly affecting SirT2 levels. A03 has known SSRI and NMDA receptor antagonist effects; however, neither SSRI fluoxetine (Prozac®) nor NMDA antagonist memantine was effective in enhancing SirT1 levels. These chemical-genetic studies indicate that the mechanism by which A03 increases SirT1 protein level is unique to the structural features of A03. We separated the enantiomers of A03, and determined their relative *in vitro* potency and *in vivo* brain-penetrance compared to A03. For *in vivo* efficacy studies, we used the ‘E4FAD’ murine model resulting from crossing 5XFAD^28^ mice to a targeted-replacement ApoE4 model^29^. After initial pilot studies with A03 in the E4FAD mouse model using subcutaneous injection of compounds, we performed longer term oral dosing studies wherein we found that 56-day treatment with A03 increased SirT1 in the hippocampus of AD model mice and improved memory as determined in the Novel Object Recognition (NOR) testing paradigm.

## Results

### HTS and validation show A03, but not memantine or fluoxetine, enhances SirT1

**Figure 2:**
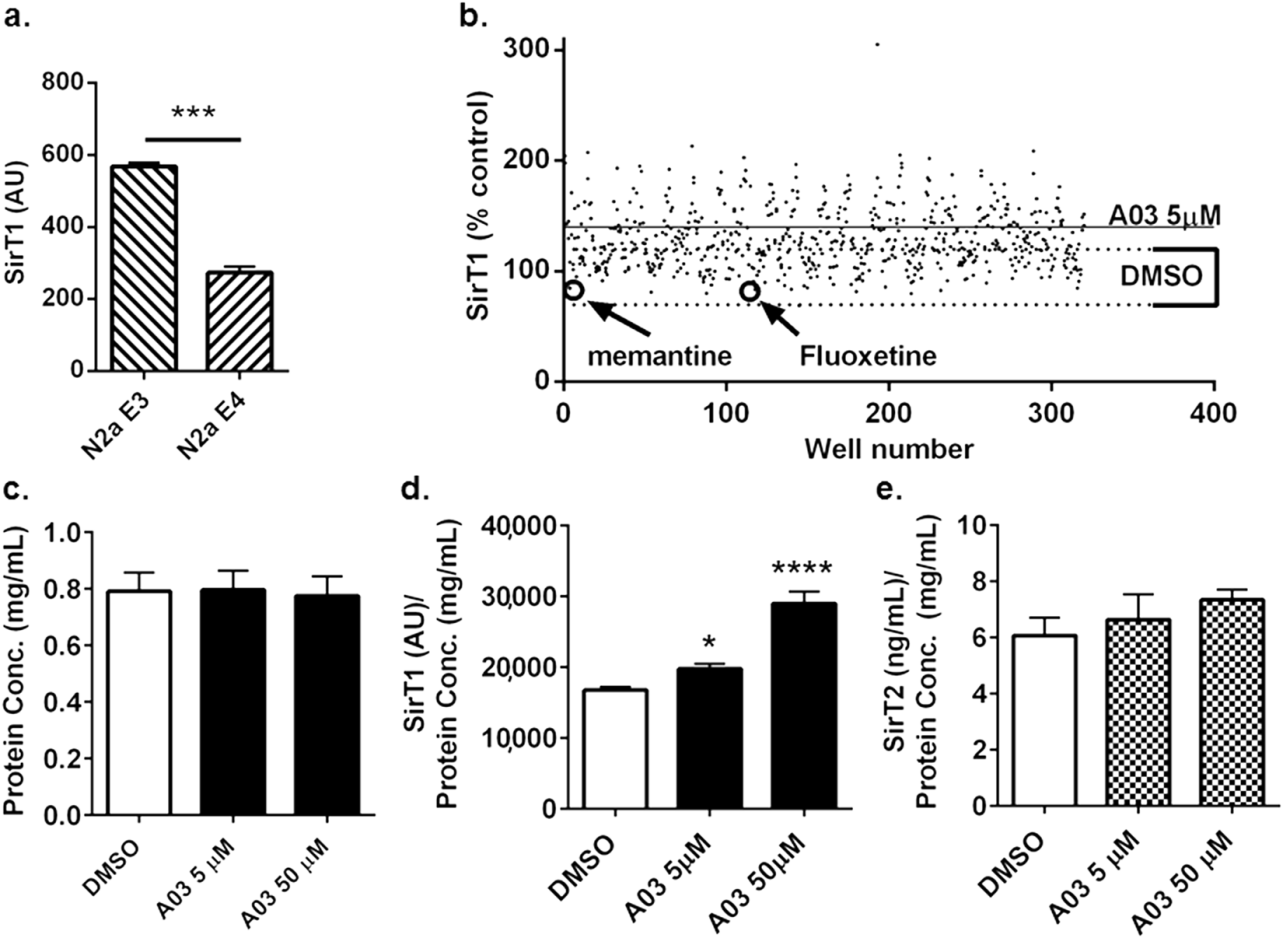
HTS of the NIH Clinical Collection module in N2a-E4 cells and effects of A03, fluoxetine, and memantine. (**a**) SirT1 levels in N2a cells stably transfected with ApoE4 (N2a-E4) are approximately half those in N2a cells stably transfected with ApoE3 (N2a-E3). (AU = Arbitrary Units; n = 3 wells per condition; *p* = 0.0001; statistical analysis performed using an unpaired two-tailed t-test). (**b**) As part of optimization and refinement of our HTS, we used N2a-E4 cells to screen the NIH Clinical Collection module comprising ~720 compounds and including multiple representations of A03 (32 per plate; −3 plates total). The mean value of SirT1 levels after A03 treatment is represented by the horizontal line, and mean value of SirT1 levels after DMSO control treatment is within the bracketed dotted lines. A03, but not SSRI fluoxetine or NMDA receptor antagonist memantine (each n = 1), increased SirT1 in N2a-E4 cells over control levels; however, because fluoxetine and memantine were only represented once in HTS, a secondary assay was performed to confirm these findings. (**c**) Protein concentrations in lysates used for assay from N2a cells treated with DMSO vehicle or A03 at 5 or 50 μM were very similar. (**d**) SirT1 (normalized to protein) showed a dose-response increase with A03 treatment (*p* = 0.0443 and <0.0001 for 5 and 50 μM, respectively). Statistical analysis was performed using one-way ANOVA and Tukey’s multiple comparison test; for the ANOVA summary F = 58.47 and *p* = < 0.0001. (**e**) SirT2 (normalized to protein) was not significantly altered by A03; the ANOVA summary was 0.8345 and *p* = 0.4651 (For protein, SirT1 and SirT2, n = 12, 3, and 3 for DMSO, 5 and 50 μM A03, respectively). Results graphed as mean ± SEM.

Murine neuroblastoma N2a cells stably transfected with ApoE4 (N2a-E4) show significantly lower levels of SirT1 as compared to cells transfected with ApoE3 (Fig. 2a), and therefore N2a-E4 cells were adopted for use in HTS. We used a customized AlphaLISA assay developed in our lab (Supplementary Fig. S3) to determine SirT1 protein levels in HTS and screened the ~720-compound NIH Clinical Collection module that contained the known SSRI fluoxetine (marketed as Prozac®) and known NMDA receptor antagonist memantine (marketed as Namenda®) and found that while A03 increased SirT1, neither fluoxetine nor memantine enhanced SirT1 in the presence of ApoE4 (Fig. 2b).

### A03 dose-response and testing in secondary screening

**Figure 3:**
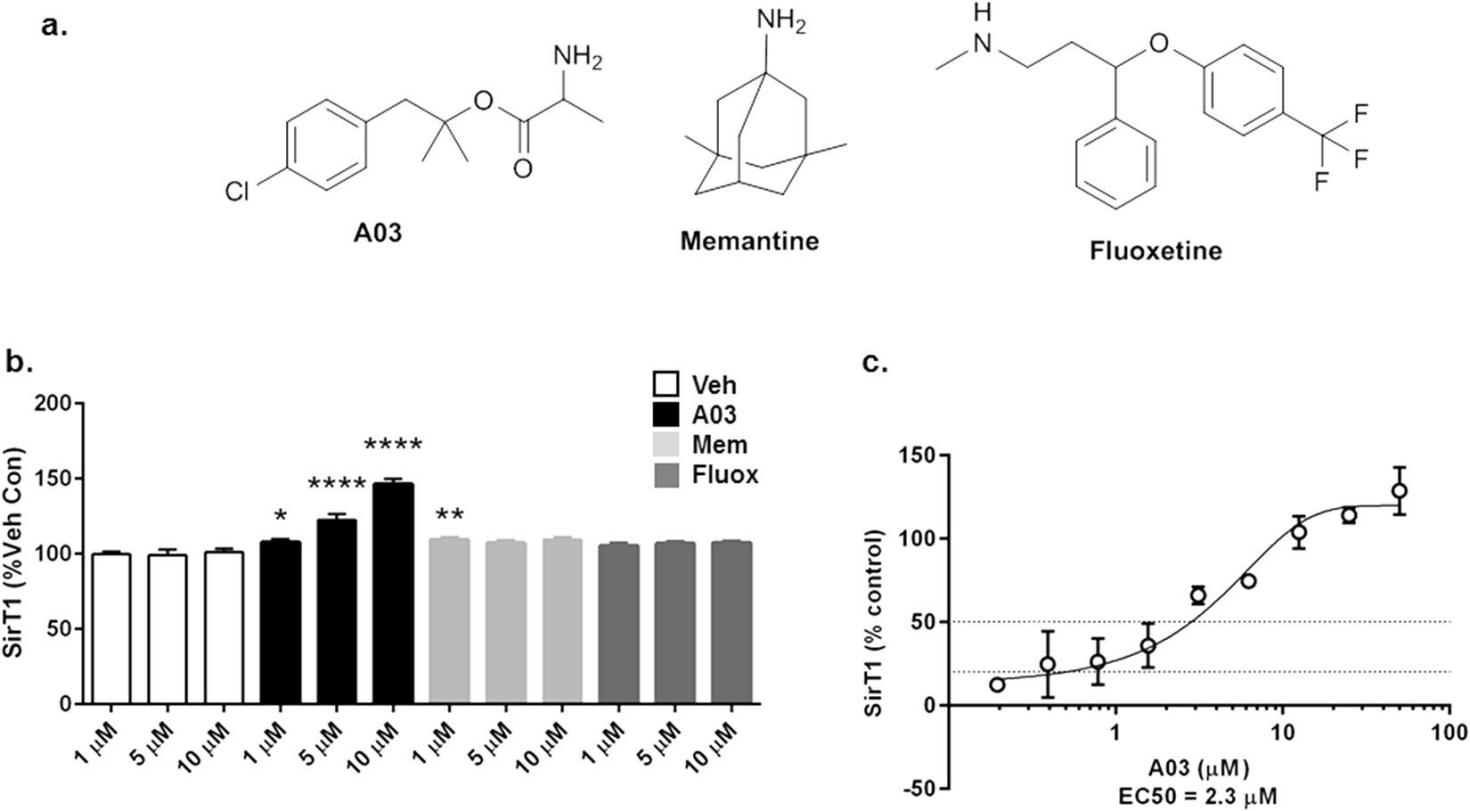
A03, fluoxetine, and memantine secondary and dose-response testing. (**a**) The structures of A03, memantine, and fluoxetine are shown. (**b**) In a secondary assay with A03, fluoxetine, and memantine at 3 different concentrations of 1, 5, and 10 μM in N2a-E4 cells, only A03 and memantine at 1 μM elicited modest but significant increases in SirT1 as compared to DMSO-only control for 1 μM (n = 6 wells per condition; *p* = 0.0184 and 0.0044, respectively). Only A03 increased SirT1 at 5 and 10 μM (*p* < 0.0001 for both) as compared to the control for each of those concentrations and showed a dose-response. All statistical analysis performed using one-way ANOVA for each compound at the same concentration level and Dunnett’s Multiple Comparisons test to compare compounds to control. The ANOVA summary at 1 μM was F = 5.058 and *p* = 0.0091; at 5 μM F = 11.15 and *p* = 0.0002; and at 10 μM F = 71.18 and *p* < 0.0001. (**c**) A dose-response curve for A03-elicited increases of SirT1 in N2a-E4 cells was used to calculate an EC50 of 2 μM (n = 3 at each concentration). Results graphed as mean ± SEM.

A03 treatment did not increase protein concentration (Bradford assay) in cell lysates (Fig. 2c), suggesting it does not affect viability under the study conditions. A03 induced dose-response increases in neuroprotective SirT1 (Fig. 2d), but not neurotoxic SirT2 (Fig. 2e) indicating the effect is selective for SirT1. A03 likely increases SirT1 protein by increasing transcription, which was reported as an increase in SirT1 mRNA in our original studies^14^. In secondary testing, neither fluoxetine nor memantine (Fig. 3a) elicited a dose-response increase in SirT1 (Fig. 3b). Memantine did increase SirT1 at 1 μM, but not at 5 or 10 μM. The dose-response enhancement of SirT1 by A03 in N2a-E4 cells gave an EC50 of 2 μM (Fig. 3c).

### A03 increases SirT1 and soluble amyloid precursor protein α (sAPPα) levels in A172-E4 cells

Our laboratory has a continuing interest in enhancement of sAPPα levels due to the neurite-supporting pro-cognitive effects of sAPPα and the relationship between these levels and the trophic balance in the brain^30,31^. To ascertain ApoE allelic form and A03 effects on sAPPα *in vitro*, we used transiently-transfected human glioblastoma cells because they express human APP and enzymes. Transfection with either ApoE3 or ApoE4 decreased SirT1 in human A172 cells as compared to empty vector, and the decrease was greater with ApoE4; A03 significantly increased SirT1 levels in A172-E4 cells (Supplementary Fig. S1). Interestingly, there was only a decrease in mean values for sAPPα with ApoE3 transfection as compared to control, and no decrease with ApoE4. Nonetheless, A03 increased sAPPα in A172-E4 cells (Supplementary Fig. S1). This suggests that the relationships between SirT1 and sAPPα, at least in the system used, is complex and requires further investigation. The increases of both SirT1 and sAPPα in this second *in vitro* model further supported advancement of A03 to *in vivo* testing.

### Enantiomer 1 (E1) elicits a greater SirT1 increase than enantiomer 2 (E2)

**Figure 4:**
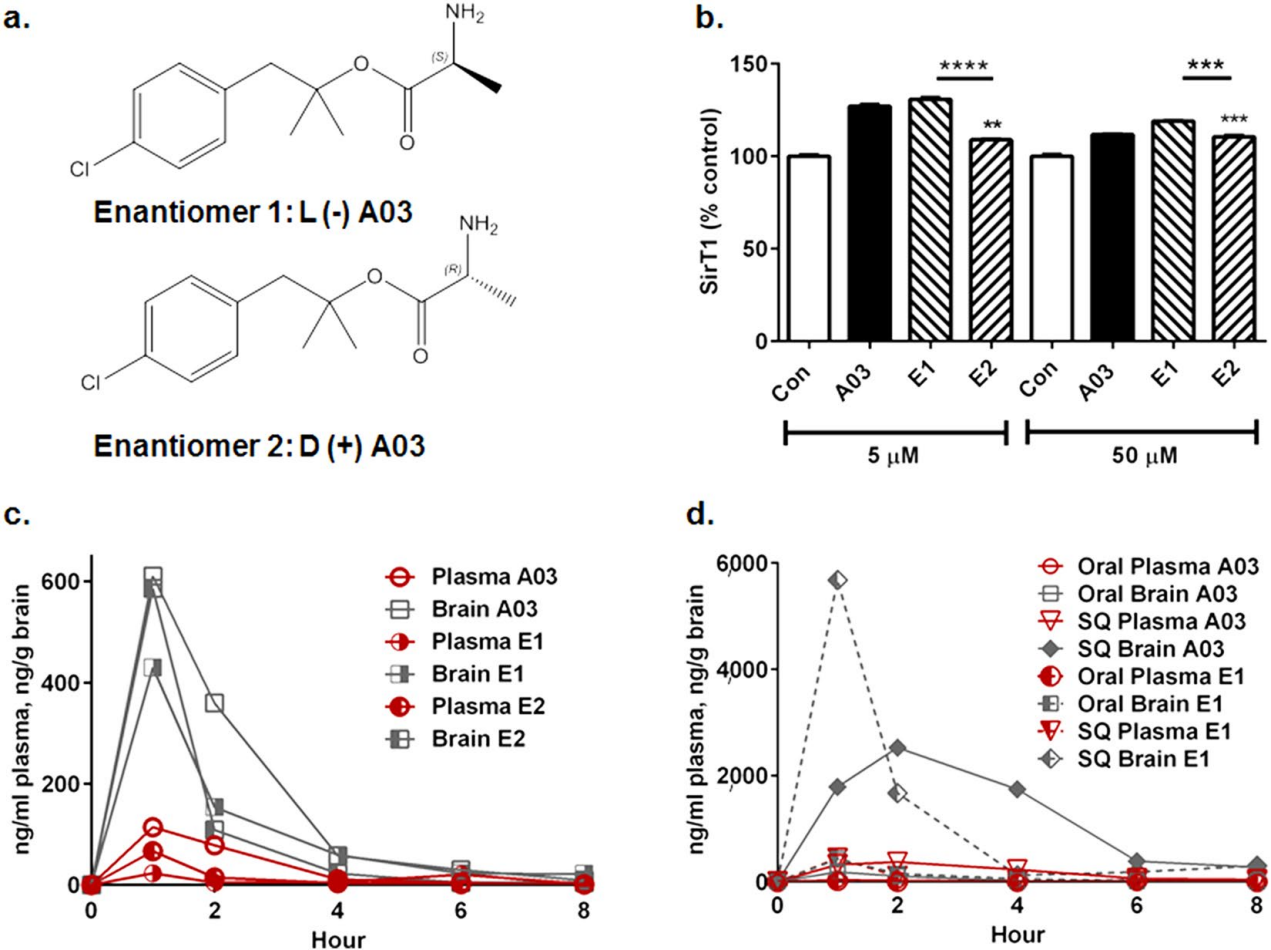
A03 and E1 have similar potency *in vitro*, but different pharmacokinetics. (**a**) The L-(−) enantiomer 1 (E1) and D-(+) enantiomer 2 (E2) are shown. (**b**) A03 and E1 increase SirT1 in N2a-E4 with high significance at both 5 and 50 μM (n = 3 wells per condition; both *p* < 0.0001, stars not shown), but E2 shows lower potency at both 5 and 50 μM (*p* = 0.0024 and 0.0002, respectively). In key comparisons (stars), E1 shows greater potency than E2 at both 5 and 50 μM (*p* < 0.0001 and *p* = 0.0009, respectively). Statistical analysis performed using one-way ANOVA and Tukey’s multiple comparisons test to compare each compound to other compounds at the same concentration. The ANOVA summary at 5 μM was F = 169.6 and p < 0.0001; and at 50 μM was F = 71.94 and p < 0.0001. Data graphed as the mean ± SEM. (**c**) After oral delivery of A03, E1 or E2 at 10 mg/kg to mice, brain levels peaked (T_max_) at 1 hour post-dose for all three compounds. The C_max_ was 611, 430, and 586 ng/g in brain and 114, 23.6, and 67 ng/ml in plasma for A03, E1 and E2, respectively. AUCs for plasma were 266, 86, and 104 ng-hr/mL and in brain were 1336, 850, and 806 ng-hr/g for A03, E1, and E2, respectively, with the caveat that in these studies, n = 1 animal per time point. After collection of blood, all animals were perfused with saline to remove residual blood from brain tissue before analysis. (**d**) After subcutaneous (SQ) injection at 10 mg/kg (n = 1; note that levels and therefore the scale is much greater for SQ than oral delivery) E1 had a shorter T_max_ (one hour) and higher C_max_ (~5600 ng/g) than A03 (C_max_ of ~2600 ng/g; T_max_ of 2 hours). The AUC for A03 in brain (10,109 ng-hr/g) was greater than that for E1 (9,101 ng-hr/g); and the plasma AUC for A03 (1515 ng/hr/mL) was more than twice that for E1 (657 ng-hr/mL). Data graphed as the mean ± SEM.

A03 is a racemate and therefore we separated the enantiomers using a chiral column and tartrate salt resolution (Supplementary Fig. S4) for determination of their relative potency and pharmacokinetic (PK) parameters. E1 (the ‘L’ enantiomer) (Fig. 4a) was found to be more effective in increasing SirT1 *in vitro* compared to the optical antipode E2 (the ‘D’ enantiomer). The SirT1 enhancing activity was similar for A03 and E1 *in vitro* in the N2a-E4 model (Fig. 4b).

### E1 and E2 give similar results in the Parallel Artificial Membrane Permeability Assay (PAMPA)

PAMPA – an *in vitro* method to test passive permeability across an artificial membrane^32^ was used to compare racemate A03 to E1 and E2. For A03, E1, and E2, respectively, retention times (T_r_) were 3.31, 3.3 and 3.31; the K_IAM_ values were 1.67, 1.66, and 1.67; and the (K_IAM_/MW^4^) * 10^−(10)^ values were 3.90, 3.88, and 3.90; indicating all three show almost identifical permeability in PAMPA.

### Oral administration of A03 has higher C_max_ and exposure than oral E1 in PK studies

A03 and both enantiomers underwent PK analysis in mice by oral delivery of each compound at 10 mg/kg, and collection of plasma and brain tissue for compound level analysis at 1, 2, 4, 6, and 8 hours after dosing. The T_max_ for all three was 60 minutes, and the C_max_ was 611, 430, and 586 ng/g in brain tissue for A03, E1, and E2, respectively (Fig. 4c). Brain levels of A03 declined more slowly than either E1 or E2, with the AUCs for brain being 1336, 850, and 806 ng-hr/g for A03, E1 and E2, respectively, indicating greater exposure for the racemate. Plasma AUCs were lower than brain AUCs in all cases, being 266, 86, and 104 ng-hr/mL for A03, E1, and E2, respectively. After subcutaneous (SQ) delivery, brain E1 level peaked at higher concentration in brain (~5800 ng/g) than A03 (~2600 ng/g) but cleared more rapidly; the plasma AUC for E1 was 657 ng-hr/mL, less than half that of A03 (1515 ng-hr/mL) and brain AUCs were 10109 and 9101 ng-hr/g for A03 and E1, respectively (Fig. 4d). It is not unexpected that the PK values differ for the racemate and the individual enantiomers. Many factors affect these values including metbolism in individual mice, relative permeability of each enantiomer across the gut (for oral delivery) and across blood-brain barrier - which may not be favorable in both cases for the same enantiomer. In addition, enantiomers may compete for mechanisms involved in uptake, distribution and clearance.

### SQ administration of A03 increased SirT1 in male E4FAD mice in the pilot efficacy study

**Figure 5:**
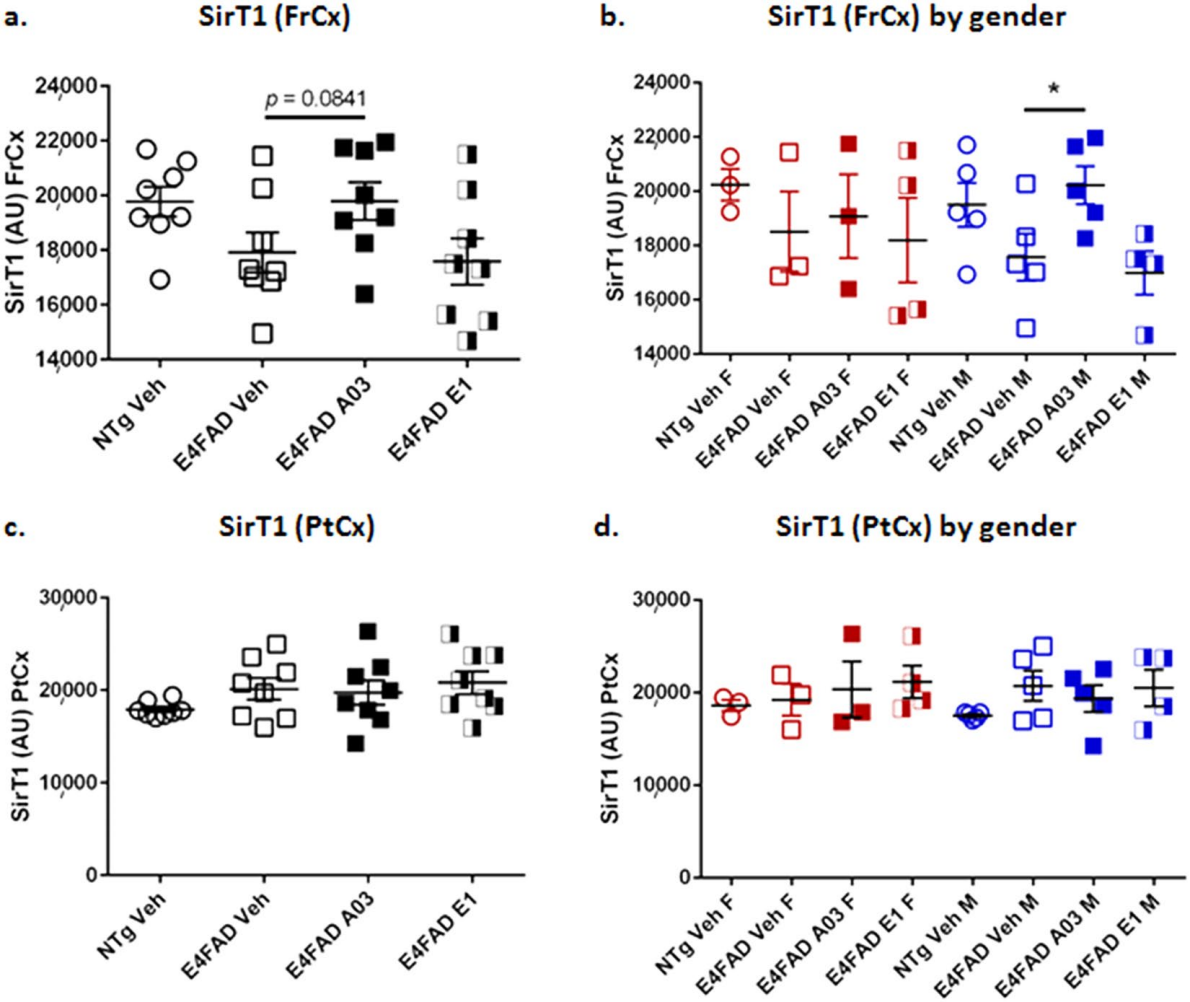
A03 by SQ delivery increases SirT1 in frontal cortex of male E4FAD mice. (**a**) After 28-day treatment of E4FAD mice with 10 mg/kg/day A03 or E1 by SQ injection, there was a trend (*p* = 0.0841) for an increase in SirT1 in the frontal cortex (FrCx) when all mice were analyzed together, but SirT1 levels were unchanged in mice treated with E1. In the ANOVA summary, F = 0.1958 and *p* = 0.8236. (**b**) When SirT1 levels in FrCx were analyzed by gender, the A03-treated male E4FAD mice showed a significant increase in SirT1 (*p* = 0.0424). Because there were fewer females, that group was likely under powered for statistics (n = 3 for NTg Veh, E4FAD Veh and A03 females, and n = 4 for E4FAD E1 females; n = 5 for all males in all groups except the E1 group, wherein n = 4). The ANOVA summary was F = 0.0833 and *p* = 0.9210 for females and F = 4.700 and *p* = 0.0335 for males. (**c**) There was no significant increase in SirT1 in the parietal cortex (PtCx) of A03 or E1-treated mice as compared to E4FAD Veh mice (AU = Arbitrary Units; n = 8 for all groups). In the one-way ANOVA summary, F = 0.1958 and *p* = 0.8236. (**d**) There was also no significant difference as a result of treatment when data were analyzed by gender. In the ANOVA summary, F = 0.2039 and *p* = 0.8202 for E4FAD females and F = 0.1990 and *p* = 0.8224 for E4FAD males (NTg mice were not compared). All statistical analysis was performed using one-way ANOVA with Tukey’s Multiple Comparison Test to compare E4FAD groups. Data graphed as the mean ± SEM.

In the *in vivo* pilot proof-of-concept efficacy study, E4FAD AD model mice were injected SQ with A03 or E1 at 10 mg/Kg/day for 28 days. Biochemical analysis revealed that there was a trend to a significant increase in SirT1 levels with A03 treatment in the frontal cortex (FrCx), as shown in Fig. 5a. The A03-induced increase was significant when males and females were analyzed separately (Fig. 5b) – a secondary analysis we typically perform because it has been reported that the expression of ApoE4 affects males and females differently^33,34^. Because there were fewer females treated with A03 than males, the former group may have been under powered for statistics. There was no significant increase in SirT1 in the parietal cortex (PtCx) with either A03 or E1 as compared to E4FAD vehicle control whether the data were analyzed as a group (Fig. 5c) or by gender (Fig. 5d). Treatment with E1 did not increase SirT1 in this study (Fig. 5a–d). FrCx and PtCx were the only brain regions analyzed in this study. In Lattanzio *et al*.^12^, SirT1 mRNA was only found to be decreased in the FrCX of E4 targeted-replacement mice and in the E4FAD mice used here, the 5XFAD transgene is under control of the Thy1 promoter and thus we did not assume the pathology would exactly recapitulate that found in humans, wherein SirT1 decreases were seen in parietal cortex.

The apparent lack of E1 efficacy in this study, despite similar increases in SirT1 in cell models, may be due to rapid clearance of E1 as seen in the PK analysis. A03 levels were slightly higher at the end of the study two hours after the last dose on the last day (time of euthanasia) as shown in Supplementary Fig. S2, and this is similar to the results at 2 hours in the SQ PK (although E1 levels are greater at one hour); the PK study also indicates a slower decline in A03 brain levels compared to E1 (Fig. 4d).

### Oral administration of A03 shows a trend to increase SirT1 in hippocampus of E4FAD mice in a 25-day study

Due to the lack of apparent efficacy of E1 in the pilot study, for subsequent efficacy studies we only tested A03 treatment compared to vehicle. Because our objective is to advance an orally available compound, in the second *in vivo* study, E4FAD mice received A03 orally at 20 mg/Kg BID (40 mg/Kg/day) for 25 days. This dose was higher than that used with SQ delivery because brain levels were lower after oral delivery in PK (C_max_ ~ 611 ng/g, ~2.4 μM at 10 mg/Kg) but above the EC50 for increase in SirT1 levels in N2a cells. In comparison after SQ treatment (C_max_ ~ 2600 ng/g ~ 10 μM at 10 mg/Kg) the A03 levels in the brain were higher by a factor of ~4 to 5 (Fig. 4c,d). BID delivery was used to maximize exposure based on indication from the pilot study that sustained exposure, rather than a high C_max_ level was likely to elicit efficacy. In this study, PtCx, FrCx and hippocampus (Hip) were collected from individual mice for analysis because the hippocampus is affected in AD, and specifically in the 5XFAD model of AD^28,35^.

**Figure 6:**
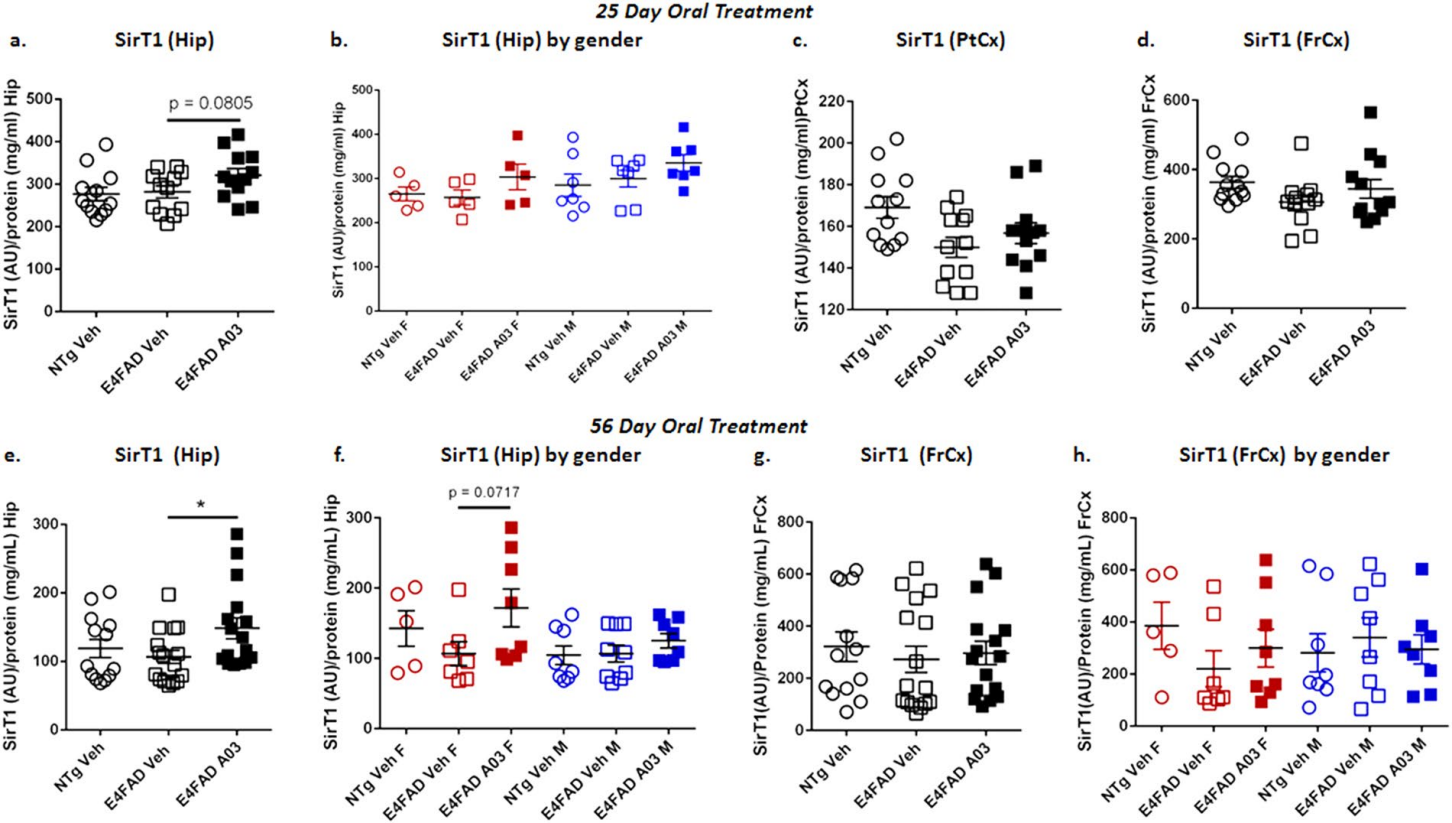
A03 shows a trend to increase SirT1 in 25-day oral study and increases SirT1 in Hip in 56-day study. (**a**) With 25-day oral dosing, there was a trend (*p* = 0.0805) for SirT1 to be increased by A03 treatment in the Hip (AU = Arbitrary Units; n = 12 for all groups). (**b**) Analysis by gender in the Hip did not reveal additional significance (n = 5 for females; n = 7 for males). (**c**) The increase in SirT1 in PtCx of E4FAD mice treated with A03 was not significant. (**d**) The results for FrCx were similar to those for PtCx. (**e**) In the longer 56-day study, there was a significant increase in SirT1 in the Hip with A03 (*p* = 0.0275; n = 13 for NTg Veh and n = 16 for E4FAD Veh and A03). (**f**) Analysis by gender showed the means for SirT1 in the Hip of both female and male A03-treated mice were higher than those for vehicle-treated mice and there was a trend to an increase (*p = *0.0717) in the females. (**g**) There was no significant increase in SirT1 in the FrCx of A03-treated E4FAD mice. (**h**) Analysis by gender did not reveal further significance. All statistical analysis performed using two-tailed, unpaired t-tests for head-to-head comparisons of Veh- and A03-treated E4FAD mice only. Data graphed as the mean ± SEM.

Only a slight increase in SirT1 in PtCx and FrCx was seen in E4FAD mice in this study with A03 as compared to vehicle-only (Fig. 6a,b), and was seen in both males and females (data not shown). Results were more promising in the Hip, where a trend (*p* = 0.0805) to an increase in SirT1 was seen with A03 treatment of E4FAD mice versus vehicle (Veh)-treated E4FAD mice (Fig. 6a); the means for SirT1 with A03 treatment were higher for both males and females (Fig. 6b). Plasma and brain levels for A03 2 hours after the last dose are shown in Supplementary Fig. S2.

### Oral A03 treatment significantly increases SirT1 in hippocampi of E4FAD mice in the 56-day study

While there was no significant increase in SirT1 in the FrCx of E4FAD mice as a result of 56-day oral treatment with A03 by either total group or gender analysis (Fig. 6e,f), there was a significant increase in SirT1 levels in the Hip as a result of A03 treatment (Fig. 6e). In this study, SirT1 increases with A03 were a little higher in females (Fig. 6f). PtCx was also analyzed and showed no significant increases in SirT1 with A03 treatment.

### No adverse effects on APP-derived biomarkers were seen with 56-day oral A03 treatment

**Figure 7:**
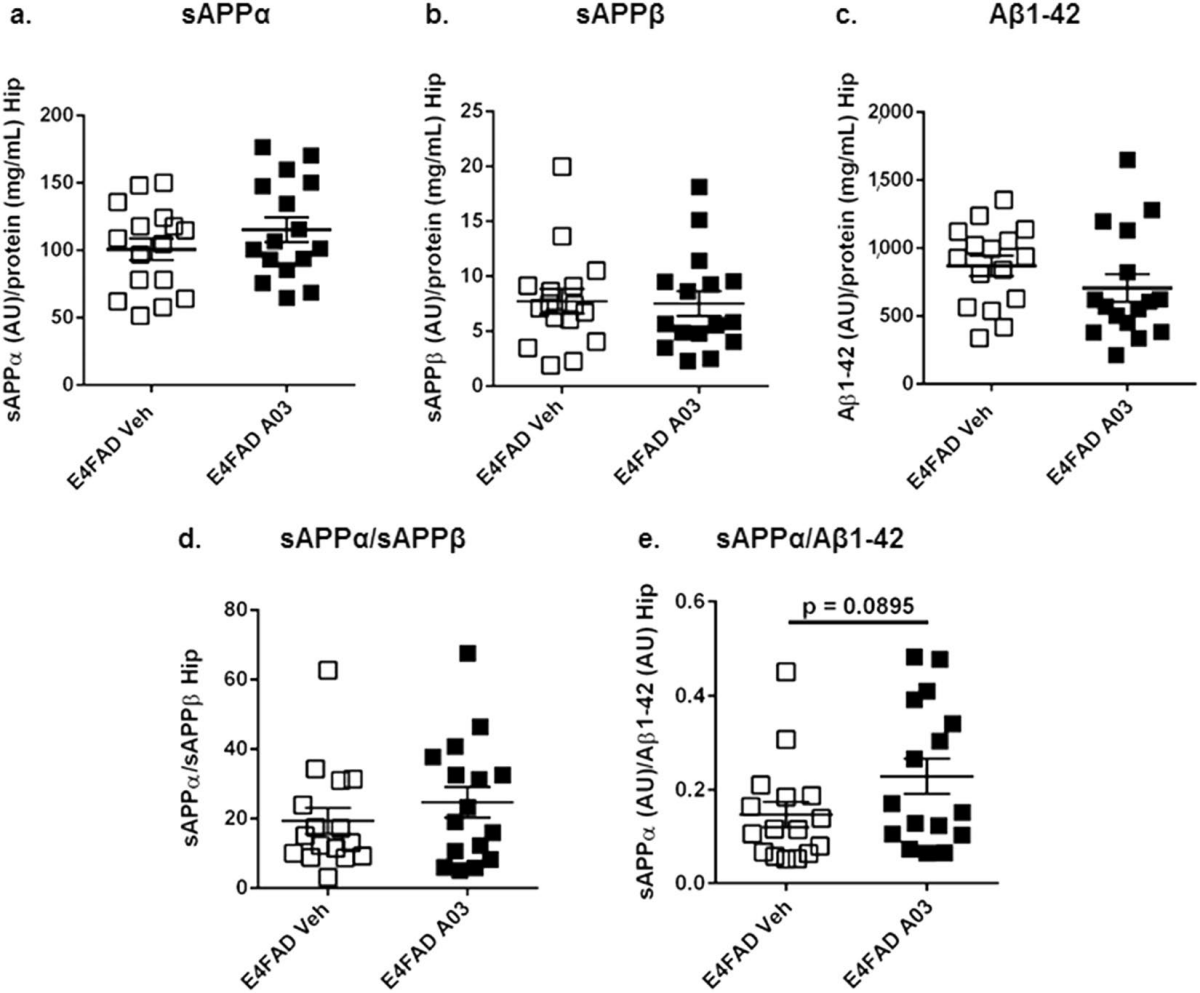
APP cleavage products after 56-day treatment of E4FAD mice. (**a**) The mean for sAPPα in the Hip of A03-treated E4FAD mice was only slightly higher than that for vehicle-treated mice (AU = Arbitrary Units). (**b**) sAPPβ was not changed in the Hip by A03 treatment. (**c**) The mean for Aβ1-42 in the Hip of A03-treated mice was lower than E4FAD Veh, but not significantly so. (**d**) There was a very modest difference in the means for the sAPPα/sAPPβ ratio in the Hip. (**e**) There was a trend (*p* = 0.0895) for an increase in the sAPPα/Aβ1-42 ratio in A03-treated mice. All statistical analysis was performed using a two-tailed, unpaired t-test to compare E4FAD groups only. All data graphed as the mean ± SEM.

The levels of APP-derived cleavage products sAPPα, sAPPβ, Aβ1-42, and the sAPPα/sAPPβ in the Hip were not significantly altered by A03 treatment (Fig. 7a–d, respectively). However, the means for sAPPα and the sAPPα/sAPPβ ratio were slightly higher in A03-treated mice, and the means for sAPPβ and Aβ1-42 slightly lower, suggesting A03 treatment was not having an adverse, β pathway-increasing effect on APP processing. This was further supported by the trend for an increase in the sAPPα/Aβ1-42 ratio in A03-treated mice (Fig. 7e).

Brain levels of A03 were found to be lower in the 56-day study (Supplementary Fig. S2) at 2 hours after the last dose as compared to the 25-day study (Supplementary Fig. S2); therefore, the longer drug exposure rather than the brain A03 C_max_ level could be a contributing factor producing the significant increase in SirT1 in the Hip. It should be noted plasma and brain tissues are collected 2 hours after dosing in these *in vivo* studies with the purpose of capturing a pharmacodynamic rather than a pharmacokinetic peak. Nonetheless, it is likely the extended duration of treatment produced the significant increase. In addition, treatment of a greater number of mice (12 in the 25-day study per group, 16 in the 56-day study) contributed to achieving statistical significance.

### Oral 56-day A03 treatment increased the discrimination index (DI) in Novel Object Recognition (NOR) in E4FAD mice

**Figure 8:**
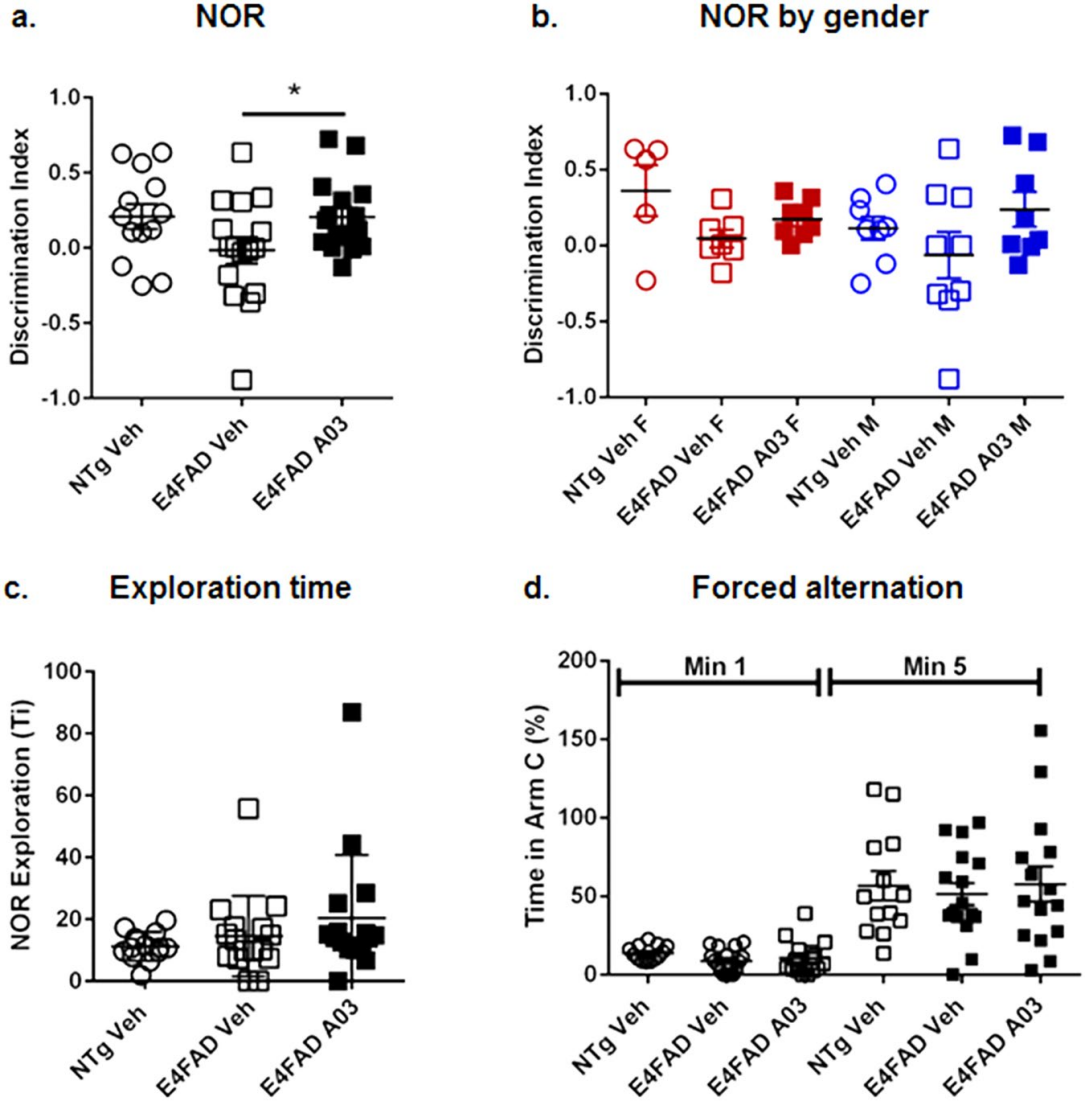
A03 improves memory. NTg Veh and Veh or A03-treated E4FAD mice underwent Novel Object Recognition (NOR) memory assessment at Day 52 and were euthanized at Day 56. (**a**) In NOR, after initial exposure to two identical objects, mice were re-exposed 24 hours later to one novel and one familiar object, and the time spent with each was recorded and analyzed by calculation of the discrimination index (DI). A positive value indicates novelty preference and a zero value no preference. A03-treated E4FAD mice showed significant novelty preference (*p* = 0.0461) as compared to vehicle-treated E4FAD controls (n = 13 for NTg Veh; n = 16 for E4FAD groups). E4FAD vehicle mice showed no novelty preference. The novelty preference of A03-treated mice was equal to that of NTg Veh mice. Because the difference between NTg Veh and E4FAD Veh mice was not significant, there may be a ceiling effect, that is, a limit to the improvement that can be seen. (**b**) While the improvement in memory in NOR did not reach significance for either gender when analyzed separately, the means for both female and male A03-treated E4FAD were higher than those for Veh-treated E4FAD mice. (**c**) Exploration time – the total time spend with both objects – was similar for E4FAD Veh and A03-treated mice, suggesting treatment did not affect activity level. (**d**) In the Y maze FA study, there were no significant differences amongst groups in entries into the Novel Arm C (divided by all arm entries) in either minute 1 or by 5 minutes. All statistical analysis was performed using a two-tailed, unpaired t-test to compare E4FAD groups only. All data graphed as the mean ± SEM.

At the end of the 56-day study, mice underwent Novel Object Recognition (NOR) memory testing. In NOR, A03-treated E4FAD mice showed novelty preference as reflected by an increase in the discrimination index (DI) (Fig. 8a). In this testing paradigm, mice are exposed to two identical objects and (in this case) after 24 hours, are exposed to one familiar and one novel object. If learning and memory are intact, a mouse will remember the familiar object and spend more time with the new object. If memory is impaired, then both objects are perceived as ‘new’ and the mice will spend equal time with each. The DI for A03-treated mice overall was equal to that of the vehicle-treated wildtype mice used in this study as the standard for memory performance. Females and males performed similarly (Fig. 8b). The exploration time – total time spend with both objects – was slightly higher for E4FAD mice as compared to NTg mice (Fig. 8c), and indicated there was no impairment in activity level in A03-treated mice. No differences were found amongst groups in the Y maze forced alternation study (Fig. 8d). We did perform an analysis to determine if there was a correlation between hippocampal SirT1 levels and performance in NOR for individual mice, but the correlation did not reach significance. It should be noted, however, that SirT1 levels are determined in tissue collected 2 hours after dosing on the last day of treatment and may not reflect the SirT1 levels in hippocampi during NOR testing performed the week previous to euthanasia.

### No signs of toxicity were seen with oral 56-day A03 treatment

As described above, there was no impairment in behavior of A03-treated mice in the 56-day study. In addition, no signs of toxicity were seen in A03-treated mice by cage-side observation, and body weights stayed the same in all groups over the course of the study (Supplementary Fig. S2).

## Discussion

We describe here for the first time an ApoE4-targeted potential therapeutic candidate for AD that reverses the deficits in brain levels of the neuroprotective NAD-dependent deacetylase SirT1. The increase of SirT1 levels in the hippocampi of the ApoE4-expressing AD model mice after oral A03 treatment for 56 days was significant and was associated with an improvement in memory. Treatment of E4FAD mice with A03 did not result in weight loss (Supplementary Fig. S2) or other adverse events. Interestingly, SirT1 levels in other brain regions were not significantly altered as compared to vehicle-treated E4FAD controls, and while we did not see a significant increase in sAPPα as a result of treatment, we attribute this to the influence of the APP and PS1 mutations in the model that by design strongly direct APP processing to the amyloidogenic pathway. In contrast, increased SirT1 levels may likely affect levels of acetyl tau and the tauopathy pathway as previously reported^14,36^. Taken together, the data presented here support the advancement of A03 as a preclinical candidate for development as the first ApoE4-targeted brain SirT1 enhancer.

A03 was originally developed as an SSRI^37^ and potential treatment for depression by the Swedish pharmaceutical company Astra AB (now AstraZeneca). Our data suggests that SirT1 enhancement is not a general activity of this pharmacological class as we only see enhancement of SirT1 with A03 and not with fluoxetine. A03 is also described as a non-competitive NMDA receptor antagonist^38,39^ and testing of the partial NMDA antagonist memantine did not show an increase in SirT1 levels as seen with A03. Thus, NMDA receptor antagonism alone does not appear to explain the SirT1 effect, since the limited reports on the impact of NMDA receptor antagonism on SirT1 have been mixed and model-dependent^40,41^. Together these data suggest that an as-yet unidentified mechanism maybe involved in the SirT1 increases seen with A03. A03 was studied in Phase 1 trials for depression, and while in one study some liver function changes were seen^42^, the effects were not significant enough for the subjects to be taken off the drug and side effects improved before the end of the trial. In a second clinical study, A03 treatment showed no toxicity up to 200 mg BID^43^. This is greater than the Human Equivalent Dose (HED) used here (~200 mg/day/human). A03 also received some attention as a potential treatment for dementia^37,44–46^. In a small clinical studies in moderate to advanced AD patients, A03 elicited some improvements in global scores^44,46^. The observed clinical improvements with A03 in these studies could have been limited by the advanced state of disease in the patients treated and no meta-analysis of the data from ApoE4 patients were reported.

As a result of our studies with A03 we have learned a great deal about the approach to testing SirT1 enhancers, including which model to use – we had previously tested ApoE4 TR mice not crossed to the 5XFAD – and the impact of age of the mice. The mice used in the studies reported here are much older than those used in our early pilot studies. The route and timing of treatment were also modified as a result of our initial studies. We wanted an orally available compound and found that oral delivery twice a day provided a level and duration of exposure that resulted in efficacy. We also found that A03 oral treatment reverses the SirT1 deficits in the hippocampus, one of the brain regions most affected in AD. It is interesting to note that A03 effects have also been described as being more pronounced in the hippocampus^37^, therefore it could have been a combination of greater disease effects and efficacy of A03 in the hippocampus that allowed us to see the increase in SirT1.

It is perhaps not surprising that a decrease in a master metabolic regulator such as SirT1 is implicated in AD and impacted by expression of a major risk factor for AD, ApoE4. The sirtuins influence aging and govern a myriad of metabolic and stress-tolerance functions^47^ and enhancement of SirT1 as an anti-AD strategy is supported by the literature. Nuclear SirT1 regulates the expression of genes, including those for FOXO3^36,48^; NFκB^49^, p53^50^, and PGC1α^51^ to trigger resistance to metabolic, oxidative, heat, and hypoxic stress^52,53^ (Fig. 1). SirT1 is reported to prevent microglia-dependent Aβ toxicity through inhibition of NFκB signaling^17^. SirT1 mediates at least some of the effects of caloric restriction (CR)^54^ and exercise, and both CR and exercise have been shown to attenuate Aβ deposition or increase Aβ clearance in murine AD models^55,56^. In primary astrocytes, SirT1’s deacetylase activity affects several proteins important for lysosomal function, and enhances lysosomal clearance of Aβ^57^. Furthermore, activation of SirT1 provides a protective cellular response by activation of the retinoic acid receptor-β (RARβ) protein and increasing transcription of the ADAM10 gene^58^ and thus α processing. Increases in α processing of both APP and Notch 1^59^ support synaptic survival and neurogenesis^59,60^. In on our ongoing pre-clinical studies of A03 we will more specifically determine effects on the SirT1 activities as described above.

Perhaps one of the most important relationships between SirT1 function and the risk for and progression of AD and related tauopathies is deacetylation of tau. Very recent studies have shown that tau acetylation has emerged as a dominant post-translational modification because the acetylation sites cluster within the microtubule-binding region, impairing MT stabilization, increasing fibrillar tau aggregates, and seeding the spread of tau pathology^61,62^. In an impressive effort by Min *et al*.^15^, they crossed a variety of mouse models to ascertain the effect of SirT1 levels on tau acetylation and determined the impact of increased SirT1 on tau pathology spread by intrahippocampal injection of AAV2-SirT1 and contralateral injection of tau fibrils. They established a role for SirT1 in deacetylation of tau and abrogation of tau pathology that supports the idea that SirT1 decreases may exacerbate pathology. Furthermore, SirT1 appears to have a role in the balance of tau exon splicing^63^ via deacetylation of splicing factor SC35 and inhibition of SC35-promoted tau exon 10 inclusion. Analysis of tau acetylation was not included in our studies here because the model we used expresses only endogenous mouse tau and therefore does not display overt tau pathology. In future studies we plan to test A03 in a model that expresses human tau and perform analyses of both phospho- and acetyl- tau.

The relevance of SirT1 levels to human disease is supported by the finding that SirT1 levels are shown to be lower in AD patient parietal cortex autopsy specimens, and that these decreases show good correlation with the time course of symptoms and tau accumulation^11^. While the exact sequence of events tethering decreases in SirT1 to onset of AD symptoms remains unclear, in a first-of-a-kind study Kumar *et al*.^64^ determined there was a decline in serum concentration of SirT1 in healthy individuals with age and, in patients with Mild Cognitive Impairment (MCI) or AD, the decline is even more pronounced and greatest for the latter. This indicates that not only is SirT1 enhancement an approach for development of new AD drugs, it could be a predictive biomarker of AD in early stages and useful in clinical testing of this class of ApoE4 targeted therapeutic.

Our drug discovery approach targets ApoE4 effects on SirT1, but others have targeted ApoE4 structure in therapeutic discovery. At the Gladstone Institute, scientists have focused on a mechanism of ApoE4-induced neurotoxicity as it relates to the conformation of the ApoE4 protein as a result of a Cys to Arg substitution at position 112, rendering it subject to enhanced proteolysis to toxic fragments. This research has led to the discovery of small-molecule ApoE4 structure correctors (SCs) that modulate the protein conformation, thereby protecting it from proteolytic degradation and promoting normal function^65^. While this is a promising approach, such structure correctors are in early stages of development. Interestingly, activators of the SirT1 enzyme have advanced into clinical testing. The small molecule activator SRT2104 has been shown to be well tolerated after single and multiple oral dosing in humans. This molecule is currently being developed by Sirtris for indications involving inflammation such as psoriasis, ulcerative colitis, and Crohn’s disease^66^.

The results described here support further development of A03 as a first-in-class ApoE4-targeted therapeutic candidate for MCI/AD that increases brain SirT1 levels in the presence of ApoE4 and has the potential to halt progression of AD-like pathology. A03 shows excellent brain penetrance and promising efficacy after chronic oral treatment in a mouse model and is a good candidate to take forward in preclinical testing for AD. In addition, through our ongoing HTS assay to discover additional SirT1 enhancers, hit validation and hit-to-lead optimization efforts we plan to design and test new chemical entities (NCEs) to identify those that are more potent analogs of A03. The goal is to develop clinical candidates that target and reverse the SirT1 deficits associated with the major genetic risk factor for AD, ApoE4, and to test such a candidate in MCI and AD patients who are carriers of the ApoE4 allele.

## Materials and Methods

### HTS

For HTS we tested a portion of the UCLA compound library (10 plates) including the NIH Clinical Collection library (3 plates). Complete medium (25 µL) was added to each well of 384-well plates, then library compounds were added (250 nL) followed by a suspension of 4 × 10^5^ cells/mL murine neuroblastoma N2a cells (25 μL) stably transfected with ApoE4 (N2a-E4). After incubation of the N2a-E4 cells with compounds for 24 hrs at 37 °C and 5% CO_2_, all but ~6 μL of medium were removed and AlphaLISA Lysis Buffer complemented with HALT protease/phosphatase inhibitor (20 μL, 1X final) was added to each well. To maximize lysis, the plates were frozen at −80 °C. For the AlphaLISA, lysates were thawed and vortexed for 1 min at 3000 rpm.

The AlphaLISA comprises streptavidin donor beads, biotinylated anti-SirT1 N-terminal antibody (19A7AB4), and acceptor beads conjugated to the anti-SirT1 C-terminal antibody (1F3); when the target protein is bound by both antibodies, the beads are in close enough proximity for a transfer of ^1^O_2_ and emission of a detectable signal (Supplementary Fig. S3). For the AlphaLISA, a 384-proxy plate was preloaded with AlphaLISA buffer (2 µL, 1X) with HALT (1X), then cell lysate (2 μL) was transferred to the proxy plate followed by the antibody-mix (2 μL). After incubation at room temperature for 1 hr, the donor bead mixture was added (2 µL) and incubated for an additional 30 min. The plates were then read in an Envision plate reader. All points are one replicate. The standard curve is generated by serial dilution of recombinant SirT1 to assess the linear range of the assay; study samples undergo serial dilution as well. We use A03 as a positive control in the HTS assay. The Z-value, while not ideal, was <1 for the assay (Supplementary Fig. S3).

### Secondary *in vitro* assays with A03 or A03, fluoxetine, and memantine

N2a-E4 cells were treated with A03, fluoxetine or memantine (1, 5 and 10 μM). Compound in medium (50 µL, 2X) was loaded into a 96-well plate, then 4 × 10^4^ cells were added to each well; 24 hrs later the medium was removed and cells lysed. Lysate protein concentration was determined using the Bradford assay according to the manufacturer’s instructions. SirT1 was determined in lysates using the SirT1 AlphaLISA as described above.

### Secondary assay for A03 effects on SirT2

N2a-E4 cells were treated with A03 and SirT2 levels were assessed using a sandwich ELISA (LifeSpan BioSciences, Inc.; Cat. #LS-F14543) following the manufacturer’s instructions.

### Enantiomer Isolation

We resolved the enantiomers of A03 and determined enantiomer-1 (E1) to be the L-alanine enantiomer and enantiomer-2 (E2) to be the D-alanine enantiomer. The enantiomer separation was done by tartrate salt formation to obtain sufficient amounts of the enantiomers for *in vitro*, pharmacokinetic (PK), and (for E1) *in vivo* efficacy studies. We used L(+)tartaric acid to isolate the L-enantiomer salt and D(-) tartaric acid to isolate the D-enantiomer salt, followed by neutralization of the salt with NaOH and re-crystallization to obtain the enantiomers. Enantiomer abundance (Supplementary Fig. S4) was determined by HPLC using a Chiralpak IB column. Isopropanol (0.2% diethylamine)/hexanes was used for gradient elution with 5–50% isopropanol (0.2% diethylamine) for 11 min, a Chiralpak IB column, and with sample (1 μL, 1.25 mg/mL) injection, 220 nm wavelength detection, and a flow rate of 1 mL/min. The absolute configuration of the resolved enantiomers was established by synthesis of the L- and D - alanine A03 analogs from the optically pure Boc-alanine and coupling with tertiary alcohol^67^ followed by deprotection and analysis by the Chiralpak column. The L-alanine analog co-elutes with enantiomer-1 while the D-alanine analog co-elutes with enantiomer-2. Analysis of the enantiomers and A03 in the Parallel Artificial Membrane Permeability Assay (PAMPA) was performed and is described in Supplementary Methods.

### *In vivo* pharmacokinetic analysis

All *in vivo* experiments described were carried out in strict accordance with good animal practice according to NIH recommendations. All procedures for animal use were approved by the Animal Research Committee (ARC) at UCLA and under an approved ARC protocol. Pharmacokinetic (PK) analysis^68–70^ consisted of oral delivery of compounds (10 mg/Kg) to individual mice followed by euthanasia at 1, 2, 4, 6, and 8 hours post-dose by ketamine/xylazine over-anesthesia, blood collection by cardiac puncture, and transcardial saline perfusion. Whole blood was centrifuged at 5000 rpm for 10 min. in a 5 °C centrifuge to isolate plasma and the plasma stored at −20 °C before analysis. Whole brains were collected, dissected down the midline, snap frozen on dry ice, and stored at −80 °C before analysis. The levels of the compounds in both plasma and brain tissue were determined by a quantitative LC/MS/MS methodology at Integrated Analytical Solutions (IAS, Berkeley, CA). Plasma samples were precipitated with an acetonitrile: methanol (1:1) cocktail containing an internal standard. Brain samples were homogenized directly in ethyl acetate by the liquid-liquid method. The resulting supernatant was evaporated to dryness and subjected to LC/MS/MS analysis. The T_max_, C_max_, brain-to-plasma ratios and brain levels were then calculated using PK Solutions software (SummitPK).

### Pilot A03 administration study

E4FAD mice^71^ resulting from crossing ApoE4 targeted-replacement mice^29,72^ to 5XFAD mice that co-express human APP with K670N/M671L, V717I, and I716V mutations and human presenilin 1 with the M146L and L286V mutations under control of the Thy1 promoter^28^ were used. E4FAD mice of 5–7 months of age received A03 (alaproclate HCl, DSK Biopharma), E1 (isolated as described above), or vehicle-only (Veh). All E4FAD groups were n = 8 for Veh and A03 (both 5 male/3 female) and E1 (4 male/4 female). In addition, there was a non-littermate non-transgenic (NTg) Veh group of ~5.5 mo mice (E4FAD mice do not have non-transgenic littermates), with an n = 8 (4 male/4 female). A03 and E1 were dissolved in sterile saline at 5 mg/ml for injection and a volume was given to achieve a dose of 10 mg/kg/day for 28 days based on individual mouse weights taken twice a week.

Mice were euthanized and blood/tissues collected 2 hrs after dosing on day 28 by the method described above for PK. Frontal and parietal cortices (FrCx and PtCx, respectively), as well as the remaining brain tissue, from individual mice were dissected, snap frozen on dry ice, and stored at −80 °C. Plasma samples and the brain tissue minus the FrCx and PtCx (the ‘rest’) from all compound-treated mice were sent to IAS for compound level analysis as described above.

Both FrCx and PtCx underwent biochemical analysis for the determination of SirT1 and sAPPα by AlphaLISA. The sAPPα AlphaLISA is similar to the SirT1 AlphaLISA but uses anti-sAPPα antibody AF1168 and 2B3-biotinylated antibodies instead of anti-SirT1 antibodies. Individual tissues were weighed and sonicated on ice with AlphaLISA Lysis Buffer complemented with HALT to give a 10% W/V sonicate. The levels of SirT1 and sAPPα in each sample were then determined in triplicate. The standards, blanks, and samples in lysis buffer were diluted with the buffer provided in the kit and added to the assay plate. The assay was performed as described for *in vitro* studies.

### First Oral 25-Day A03 administration Study

E4FAD mice received A03 BID (20 mg/Kg; 40 mg/Kg/day) or Veh with an 8–10 hour interval between doses for 25 days. Mice were 9–14 (most >11) months of age at the start of the study and n = 12 (5 females/7 males). A03 was dissolved in propylene glycol: strawberry syrup (1:3, 25 mg/mL) for oral delivery. The method for oral delivery was not gavage, but rather oral feeding of a volume of formulation based on the individual weights of the mice to give the specified dose is introduced to the mouse’s mouth using a pipette tip; this method reduces risk of esophageal injury.

Mice were euthanized and blood/tissues collected 2 hrs after dosing on Day 25 by the method described above for PK. FrCx, PtCx, and hippocampi (Hip) were collected from all mice for SirT1 and sAPPα level determination by AlphaLISA. Protein concentration was determined using a Bradford protein assay. Brain and plasma levels of A03 were determined at IAS.

### Second Oral 56-Day A03 administration Study

Mice received A03 BID (20 mg/Kg, 40 mg/Kg/day), or vehicle-only (Veh), as above, but treatment was extended to 56 days and an end-study working object memory assay, Novel Object Recognition (NOR), was added. N numbers were increased to 16 (9 male/7 female) for the E4FAD Veh group, 16 (8 male/8 female) in the E4FAD A03 group; and 13 (8 male/5 female) for the NTg Veh group. E4FAD mice were 10–12 mo and NTg mice 7–9 mo at the start of the study.

All mice underwent NOR testing the last week of dosing. Mice were acclimated to a 30 × 40 cm arena for 15 minutes 24 hrs before acquisition. Individual mice underwent acquisition by exposure to two identical objects for 10 min. and then were returned to the home cage overnight. For the probe, individual mice were returned to the arena wherein one of the objects had been changed. Mice were allowed to explore the objects for 10 minutes. Both the acquisition and probe were recorded on tripod-mounted video cameras. The number of discreet interactions with each object (nose within 1 cm of the object) and time spent with each object were scored by personnel blind to the treatment and genotype of the mice; scores were spot-checked by a second scorer. The discrimination index (DI) was calculated by subtracting time spent (or number of interactions) with the familiar object (*T*_F_) from time spent (or number of interactions) with the novel object (*T*_N_), and then dividing that value by the sum of those two values [DI = (*T*_N_ − *T*_F_)/(*T*_N_ + *T*_F_)]^73^.

Study mice also underwent Forced Alternation (FA) tests in a Y maze apparatus^74,75^. Details of the method can be found in Supplementary Methods. Briefly, individual test mice are prevented from entering one of three arms of the maze in the first 5 minute trial and after 30 minutes is returned to maze wherein the blocked arm is available. Time in Novel Arm C [%] was defined as the time spent in the novel arm divided by the time spent in all arms during the first minute.

Mice were euthanized and blood/tissues collected 2 hrs after dosing on Day 56 by the method described above for PK. Hip and FrCx were collected for analysis of SirT1, sAPPα, and Aβ1-42 levels by AlphaLISA. For Aβ1-42 the Perkin Elmer kit AL276C was used. Protein concentrations were determined using the Bradford protein assay. The sAPPα and sAPPβ AlphaLISAs were similar to the SirT1 AlphaLISA (see Supplementary Fig. S3) but the former uses anti-sAPPα antibody AF1168 and 2B3-biotinylated antibodies and the latter an anti-sAPPβ antibody (IBL Cat. #18957) and an anti-N-terminal APP antibody (R&D Systems AF1168) instead of anti-SirT1 antibodies. Brain tissue and plasma were sent to IAS for A03 level analysis.

### Statistical analysis

All statistical analyses for *in vitro* and *in vivo* studies was performed using GraphPad Prism® software; for one-way ANOVA, with level of significance set at p < 0.05. A two-tailed unpaired Student’s t-test was utilized where appropriate, with a p < 0.05 considered significant. Note than in efficacy studies, the NTg mice are not littermates of E4FAD mice (which have no NTg littermates), therefore, for statistical analysis of treatment effects, only the E4FAD groups are compared.

## Electronic supplementary material

Supplementary Infomation

## Data Availability

The authors agree to comply with data availability requirements of the Journal in accordance with their institutions data sharing plan.

## References

[1] Kang J (1987). The precursor of Alzheimer’s disease amyloid A4 protein resembles a cell-surface receptor. Nature.

[2] Dickson DW (1997). Discovery of new lesions in neurodegenerative diseases with monoclonal antibody techniques: is there a non-amyloid precursor to senile plaques?. Am J Pathol.

[3] Walsh DM, Selkoe DJ (2004). Deciphering the molecular basis of memory failure in Alzheimer’s disease. Neuron.

[4] Racchi M, Mazzucchelli M, Porrello E, Lanni C, Govoni S (2004). Acetylcholinesterase inhibitors: novel activities of old molecules. Pharmacol Res.

[5] Farrer LA (1995). Apolipoprotein E genotype in patients with Alzheimer’s disease: implications for the risk of dementia among relatives. Annals of neurology.

[6] Liu CC (2017). ApoE4 Accelerates Early Seeding of Amyloid Pathology. Neuron.

[7] Cao J (2017). ApoE4-associated phospholipid dysregulation contributes to development of Tau hyper-phosphorylation after traumatic brain injury. Scientific reports.

[8] Mahley RW, Huang Y, Weisgraber KH (2007). Detrimental effects of apolipoprotein E4: potential therapeutic targets in Alzheimer’s disease. Current Alzheimer research.

[9] Vassar R (2017). Seeds of Destruction: New Mechanistic Insights into the Role of Apolipoprotein E4 in Alzheimer’s Disease. Neuron.

[10] Theendakara V (2013). Neuroprotective Sirtuin ratio reversed by ApoE4. Proceedings of the National Academy of Sciences of the United States of America.

[11] Julien C (2009). Sirtuin 1 reduction parallels the accumulation of tau in Alzheimer disease. Journal of neuropathology and experimental neurology.

[12] Lattanzio FCL, Carretta D, Rimondini R, Candeletti S, Romualdi P (2014). Human apolipoprotein E4 modulates the expression of Pin1, Sirtuin 1, and Presenilin 1 in brain regions of targeted replacement apoE mice. Neuroscience.

[13] Rhinn H, Qiang FR, Cheng L, Lee R, Abeliovich JH (2013). A.. Integrative genomics identifies APOE ε4 effectors in Alzheimer’s disease. Nature.

[14] Theendakara V (2016). Direct Transcriptional Effects of Apolipoprotein E. The Journal of neuroscience: the official journal of the Society for Neuroscience.

[15] Min Sang-Won, Sohn Peter Dongmin, Li Yaqiao, Devidze Nino, Johnson Jeffrey R., Krogan Nevan J., Masliah Eliezer, Mok Sue-Ann, Gestwicki Jason E., Gan Li (2018). SIRT1 Deacetylates Tau and Reduces Pathogenic Tau Spread in a Mouse Model of Tauopathy. The Journal of Neuroscience.

[16] Bredesen DE (2009). Neurodegeneration in Alzheimer’s disease: caspases and synaptic element interdependence. Molecular neurodegeneration.

[17] Chen J (2005). SIRT1 protects against microglia-dependent amyloid-beta toxicity through inhibiting NF-kappaB signaling. The Journal of biological chemistry.

[18] Bai X, Yao L, Ma X, Xu X (2018). Small Molecules as SIRT Modulators. Mini reviews in medicinal chemistry.

[19] Villalba JM, Alcain FJ (2012). Sirtuin activators and inhibitors. BioFactors (Oxford, England).

[20] Alcain FJ, Villalba JM (2009). Sirtuin activators. Expert opinion on therapeutic patents.

[21] Mokni M, Elkahoui S, Limam F, Amri M, Aouani E (2007). Effect of resveratrol on antioxidant enzyme activities in the brain of healthy rat. Neurochemical research.

[22] Ahmed T (2017). Resveratrol and Alzheimer’s Disease: Mechanistic Insights. Molecular neurobiology.

[23] Neves AR, Lucio M, Lima JL, Reis S (2012). Resveratrol in medicinal chemistry: a critical review of its pharmacokinetics, drug-delivery, and membrane interactions. Current medicinal chemistry.

[24] Ishisaka A (2011). Accumulation of orally administered quercetin in brain tissue and its antioxidative effects in rats. Free radical biology & medicine.

[25] Youdim KA, Qaiser MZ, Begley DJ, Rice-Evans CA, Abbott NJ (2004). Flavonoid permeability across an *in situ* model of the blood-brain barrier. Free radical biology & medicine.

[26] Zhou Y, Wang S, Li Y, Yu S, Zhao Y (2017). SIRT1/PGC-1alpha Signaling Promotes Mitochondrial Functional Recovery and Reduces Apoptosis after Intracerebral Hemorrhage in Rats. Frontiers in molecular neuroscience.

[27] Turner RS (2015). A randomized, double-blind, placebo-controlled trial of resveratrol for Alzheimer disease. Neurology.

[28] Oakley H (2006). Intraneuronal beta-amyloid aggregates, neurodegeneration, and neuron loss in transgenic mice with five familial Alzheimer’s disease mutations: potential factors in amyloid plaque formation. The Journal of neuroscience: the official journal of the Society for Neuroscience.

[29] Sullivan PM, Mace BE, Maeda N, Schmechel DE (2004). Marked regional differences of brain human apolipoprotein E expression in targeted replacement mice. Neuroscience.

[30] Spilman, P. J. B. Bredesen, D. E. & John, V. Enhancement of sAPPalpha as a Therapeutic Strategy for Alzheimer’s and other Neurodegenerative Diseases. *Journal of Alzheimer’s and Neurodegenerative Diseases* **1** (2015).

[31] Peters-Libeu C (2015). sAbetaPPalpha is a Potent Endogenous Inhibitor of BACE1. Journal of Alzheimer’s disease: JAD.

[32] Ong S, Liu H, Pidgeon C (1996). Immobilized-artificial-membrane chromatography: measurements of membrane partition coefficient and predicting drug membrane permeability. J Chromatogr A.

[33] Ungar L, Altmann A, Greicius MD (2014). Apolipoprotein E, gender, and Alzheimer’s disease: an overlooked, but potent and promising interaction. Brain imaging and behavior.

[34] Leung L (2012). Apolipoprotein E4 causes age- and sex-dependent impairments of hilar GABAergic interneurons and learning and memory deficits in mice. PloS one.

[35] Devi L, Tang J, Ohno M (2015). Beneficial effects of the beta-secretase inhibitor GRL-8234 in 5XFAD Alzheimer’s transgenic mice lessen during disease progression. Current Alzheimer research.

[36] Elliott PJ, Jirousek M (2008). Sirtuins: novel targets for metabolic disease. Curr Opin Investig Drugs.

[37] Ogren SO, Holm AC, Hall H, Lindberg UH (1984). Alaproclate, a new selective 5-HT uptake inhibitor with therapeutic potential in depression and senile dementia. Journal of neural transmission.

[38] Svensson BE, Werkman TR, Rogawski MA (1994). Alaproclate effects on voltage-dependent K+ channels and NMDA receptors: studies in cultured rat hippocampal neurons and fibroblast cells transformed with Kv1.2 K+ channel cDNA. Neuropharmacology.

[39] Wilkinson A, Courtney M, Westlind-Danielsson A, Hallnemo G, Akerman KE (1994). Alaproclate acts as a potent, reversible and noncompetitive antagonist of the NMDA receptor coupled ion flow. The Journal of pharmacology and experimental therapeutics.

[40] Ota H, Ogawa S, Ouchi Y, Akishita M (2015). Protective effects of NMDA receptor antagonist, memantine, against senescence of PC12 cells: A possible role of nNOS and combined effects with donepezil. Experimental gerontology.

[41] Zhu D (2017). Postnatal Administration of Dizocilpine Inhibits Neuronal Excitability in PFC and Induces Social Deficits Detected by MiceProfiler. Molecular neurobiology.

[42] Frost SJ, Eccleston D, Marshall EF, Hassanyeh F (1984). Alaproclate–an open clinical study in depressive illness. Psychopharmacology.

[43] Aberg-Wistedt A, Alvariza M, Bertilsson L, Malmgren R, Wachtmeister H (1985). Alaproclate a novel antidepressant? A biochemical and clinical comparison with zimeldine. Acta psychiatrica Scandinavica.

[44] Bergman I (1983). Alaproclate: a pharmacokinetic and biochemical study in patients with dementia of Alzheimer type. Psychopharmacology.

[45] Altman HJ, Nordy DA, Ogren SO (1984). Role of serotonin in memory: facilitation by alaproclate and zimeldine. Psychopharmacology.

[46] Dehlin O, Hedenrud B, Jansson P, Norgard J (1985). A double-blind comparison of alaproclate and placebo in the treatment of patients with senile dementia. Acta psychiatrica Scandinavica.

[47] Sinclair DA (2005). Toward a unified theory of caloric restriction and longevity regulation. Mech Ageing Dev.

[48] Brunet A (2004). Stress-dependent regulation of FOXO transcription factors by the SIRT1 deacetylase. Science.

[49] Yeung F (2004). Modulation of NF-kappaB-dependent transcription and cell survival by the SIRT1 deacetylase. EMBO J.

[50] Langley E (2002). Human SIR2 deacetylates p53 and antagonizes PML/p53-induced cellular senescence. EMBO J.

[51] Wang J (2010). The role of Sirt1: at the crossroad between promotion of longevity and protection against Alzheimer’s disease neuropathology. Biochim Biophys Acta.

[52] Guarente L (2009). Cell biology. Hypoxic hookup. Science.

[53] Bonda DJ (2011). The sirtuin pathway in ageing and Alzheimer disease: mechanistic and therapeutic considerations. Lancet Neurol.

[54] Guarente L (2008). Mitochondria–a nexus for aging, calorie restriction, and sirtuins?. Cell.

[55] Koo JH, Kang EB, Oh YS, Yang DS, Cho JY (2017). Treadmill exercise decreases amyloid-beta burden possibly via activation of SIRT-1 signaling in a mouse model of Alzheimer’s disease. Experimental neurology.

[56] Patel NV (2005). Caloric restriction attenuates Abeta-deposition in Alzheimer transgenic models. Neurobiol Aging.

[57] Li MZ (2018). SIRT1 facilitates amyloid beta peptide degradation by upregulating lysosome number in primary astrocytes. Neural regeneration research.

[58] Tippmann F, Hundt J, Schneider A, Endres K, Fahrenholz F (2009). Up-regulation of the alpha-secretase ADAM10 by retinoic acid receptors and acitretin. FASEB journal: official publication of the Federation of American Societies for Experimental Biology.

[59] Costa RM, Drew C, Silva AJ (2005). Notch to remember. Trends Neurosci.

[60] Xiao MJ, Han Z, Shao B, Jin K (2009). Notch signaling and neurogenesis in normal and stroke brain. International journal of physiology, pathophysiology and pharmacology.

[61] Min SW (2015). Critical role of acetylation in tau-mediated neurodegeneration and cognitive deficits. Nature medicine.

[62] Trzeciakiewicz H (2017). A Dual Pathogenic Mechanism Links Tau Acetylation to Sporadic Tauopathy. Scientific reports.

[63] Yin X (2018). SIRT1 Deacetylates SC35 and Suppresses Its Function in Tau Exon 10 Inclusion. Journal of Alzheimer’s disease: JAD.

[64] Kumar R (2013). Sirtuin1: a promising serum protein marker for early detection of Alzheimer’s disease. PloS one.

[65] Mahley RW, Huang Y (2012). Small-molecule structure correctors target abnormal protein structure and function: structure corrector rescue of apolipoprotein E4-associated neuropathology. Journal of medicinal chemistry.

[66] Hoffmann E (2013). Pharmacokinetics and tolerability of SRT2104, a first-in-class small molecule activator of SIRT1, after single and repeated oral administration in man. Br. J. Clin. Pharmacol..

[67] Lindberg UH (1978). Inhibitors of neuronal monoamine uptake. 2. Selective inhibition of 5-hydroxytryptamine uptake by alpha-amino acid esters of phenethyl alcohols. Journal of medicinal chemistry.

[68] Spilman P (2014). The multi-functional drug tropisetron binds APP and normalizes cognition in a murine Alzheimer’s model. Brain research.

[69] Korfmacher WA (2001). Cassette-accelerated rapid rat screen: a systematic procedure for the dosing and liquid chromatography/atmospheric pressure ionization tandem mass spectrometric analysis of new chemical entities as part of new drug discovery. Rapid Commun. Mass Spectrom..

[70] Mei H, Korfmacher W, Morrison R (2006). Rapid *in vivo* oral screening in rats: reliability, acceptance criteria, and filtering efficiency. The AAPS journal.

[71] Youmans KL (2012). APOE4-specific changes in Abeta accumulation in a new transgenic mouse model of Alzheimer disease. The Journal of biological chemistry.

[72] Knouff C (1999). Apo E structure determines VLDL clearance and atherosclerosis risk in mice. The Journal of clinical investigation.

[73] Antunes M, Biala G (2012). The novel object recognition memory: neurobiology, test procedure, and its modifications. Cognitive processing.

[74] Magen I (2012). Cognitive deficits in a mouse model of pre-manifest Parkinson’s disease. The European journal of neuroscience.

[75] Wolf A, Bauer B, Abner EL, Ashkenazy-Frolinger T, Hartz AM (2016). A Comprehensive Behavioral Test Battery to Assess Learning and Memory in 129S6/Tg2576 Mice. PloS one.

