# Supplementary material for: A small molecule ApoE4-targeted therapeutic candidate that normalizes sirtuin 1 levels and improves cognition in an Alzheimer’s disease mouse model
Source: Sci Rep. 2018 Dec 4;8:17574. doi: 10.1038/s41598-018-35687-8 (PMC6279743; doi:10.1038/s41598-018-35687-8)

Supplementary Materials

A small molecule ApoE4-targeted therapeutic candidate that normalizes sirtuin 1 levels and Improves cognition in an Alzheimer's disease mouse model

Jesus Campagna, Patricia Spilman, Barbara Jagodzinska, Dongsheng Bai, Asa Hatami, Chunni Zhu, Tina Bilousova, Michael Jun, Chris Jean Elias, Johnny Pham, Gregory Cole, Mary Jo LaDu, Michael E. Jung, Dale E. Bredesen, and Varghese John*

Supplementary Methods

Confirmation assay in A172-E4 cells. Before transfection, A172 human glioblastoma cells were cultured in Dulbecco's Modified Eagle Medium (DMEM) supplemented with 10% fetal bovine serum and 1% penicillin/streptomycin in a 37°C incubator in an atmosphere of 5% CO_2 in air. The day of transfection, the media was replaced with Opti-MEM reduced serum medium. Transient transfection with human ApoE4 and ApoE3 constructs in pcDNA3 mammalian expression vectors or empty vector (control) was performed in 96-well plates using a 1:1 mixture of two transfection reagents - Lipofectamine 2000 and siPORT amine - in Opti-MEM as previously described[13]. After 4 hr incubation with transfection reagents, medium was exchanged for fresh DMEM containing 50 µM of A03 or DMSO (0.5% final). Cells were incubated with the compound for 60 hrs, then cell culture medium (for sAPPα) and cells (lysates for SirT1) were harvested and levels of sAPPα and SirT1 determined by AlphaLISA. For the sAPPα AlphaLISA, a biotinylated anti-sAPPα N-terminal antibody (AF1168, R&D) was used for the donor beads and an anti-sAPPα C-terminal antibody (AL231C, Perkin-Elmer) for the acceptor beads.

PAMPA. An IAM.PC.DD2 Regis Technologies analytical (10 cm x 4.6 mm) column wherein phospholipid is bonded on 10 µm, 300 Å, spherical aminopropyl silica and end capped with C10 and C3 amides was used to determine membrane permeability of A03, E1 and E2. An isocratic method was used with a mobile phase comprising acetonitrile:PBS (pH 5.6) equal to 35:65, an injection volume of 5 µL, and a flow rate of 1 mL/min. The parameters determined are T_r = retention time of compound; K_{IAM}= $(T_r$-$T_0)/T_0$; T_0= is the delay time of the column, determined by use of 100 µg/mL citric acid) and was T_0= 1.24 min; and $(K_{IAM}/MW^4)*10^{-(10)}$. The molecular weight of all three compounds was 255.74 g/mol.

Forced Alternation. The test, performed using a Y maze apparatus, consisted of a 5 min sample trial (T1) followed by a 5 min retrieval trial (T2). In T1, the mouse was placed into the end of the start arm, facing the wall and away from the center. The mouse was then allowed to explore two arms of the Y-maze, while entry into the third arm (C) was blocked. After the sample trial, the mouse was returned to its home cage for a 30 min inter-trial interval. In T2, the block in arm 3 was removed, the mouse was again placed into the start arm, and then allowed to access all three arms of the maze. After each animal and between T1 and T2, the maze was wiped with a 70% ETOH to prevent odor cues. An arm entry was recorded when all 4 paws of the mouse entered the arm. Time in Novel Arm C [%] was defined as the time spent in the novel arm divided by the time spent in all arms during the first minute of the retrieval trial T2. Mice with less than three arm entries in the first minute of T2 were excluded from the analysis.

Supplementary Figure Legends

Supplementary Fig. S1. *A03 increases SirT1 and sAPPα in A172-E4 cells.* (a) Transient transfection with either ApoE3 or E4 decreased SirT1 levels as compared to empty vector (Pc/DMSO), but the decrease was greater with

ApoE4 (AU = Arbitrary Units; $p = 0.0006$ and $p < 0.0001$ for ApoE3 and E4 compared to Pc/DMSO, respectively). Twenty-four hour treatment with 50 µM A03 significantly increased SirT1 levels in ApoE4-transfected cells ($p = 0.0025$) (b) While sAPPα levels were not significantly altered by transfection, the mean was lower for ApoE3 than empty vector and ApoE4. Treatment with A03 resulted in a significant increase in trophic, pro-cognitive α-secretase full-length APP cleavage product sAPPα ($p = 0.0414$). All statistical analysis was performed using one-way ANOVA (summary for (A): $p < 0.001$ and F = 34.07; summary for (B): $p = 0.0050$ and F = 8.103) with Tukey's multiple comparison test for head-to-head comparisons. (n = 2 for Pc/DMSO, n = 3 for E3/DMSO, and n = 4 for E4/DMSO and E4/A03). Data graphed as the mean ± SEM.

Supplementary Fig. S2. *Plasma and brain levels of E1 and A03, and body weights, in vivo*. (a) Brain levels of E1 were slightly lower than brain levels of A03 2 hours after the last dose in the pilot study wherein compounds were delivered by SQ injection at 10 mg/kg/day for 28 days (n = 8). (b) In the first 25-day study of oral delivery of A03 at 40 mg/kg/day, brain levels were just above 2000 n/g 2 hours after the last dose (n = 12). (c) In the 56-day study of oral delivery of A03 at 40 mg/kg/day, A03 brain levels were closer to 700 ng/g 2 hours after the last dose (n = 16). (d) Body weights of mice in the 56-day oral A03 study remained nearly the same from pre-study/Day 1, week 4 of treatment, to end-study Day 56 for all mice, regardless of genotype or treatment. Data graphed as the mean ± SEM.

Supplementary Fig. S3. *SirT1 AlphaLISA*. (a) SirT1 is detected by binding of a biotinylated (yellow) anti-N-terminal SirT1 antibody (AB4) and an anti-C-terminal SirT1 antibody (1F3) bound to an 'acceptor' bead. The streptavidin (orange) labeled 'donor' bead binds the biotin, bringing the donor and acceptor beads in close proximity; excitation at 680 nm leads to release of oxygen radicals and emission at 615 nm. (b) The assay shows good linearity with increasing volume of analyte. The standard curve is generated by serial dilution of recombinant SirT1 to assess the linear range of the assay; study samples undergo serial dilution as well. (c) A03 5 µM significantly increased SirT1, with a n = 320 and Z' = < 1. Data graphed as the mean ± SEM.

Supplementary Fig. S4. *Isolation of enantiomers of A03*. (a) A03 (box) has a chiral center as shown by the asterisk. The racemate comprises ~1:1 mix of the enantiomers; (b) Enantiomer 1 constitutes 96 % of A03 present after separation of the *S*-enantiomer; (c) Enantiomer 2 constitutes 93.5 % of A03 present after separation of the *R*-enantiomer. The enantiomers of A03 were resolved and isolated by formation of their tartrate salts, followed by analysis using a Chiralpak HPLC column.

Supplementary Figures

Supplementary Figure S1

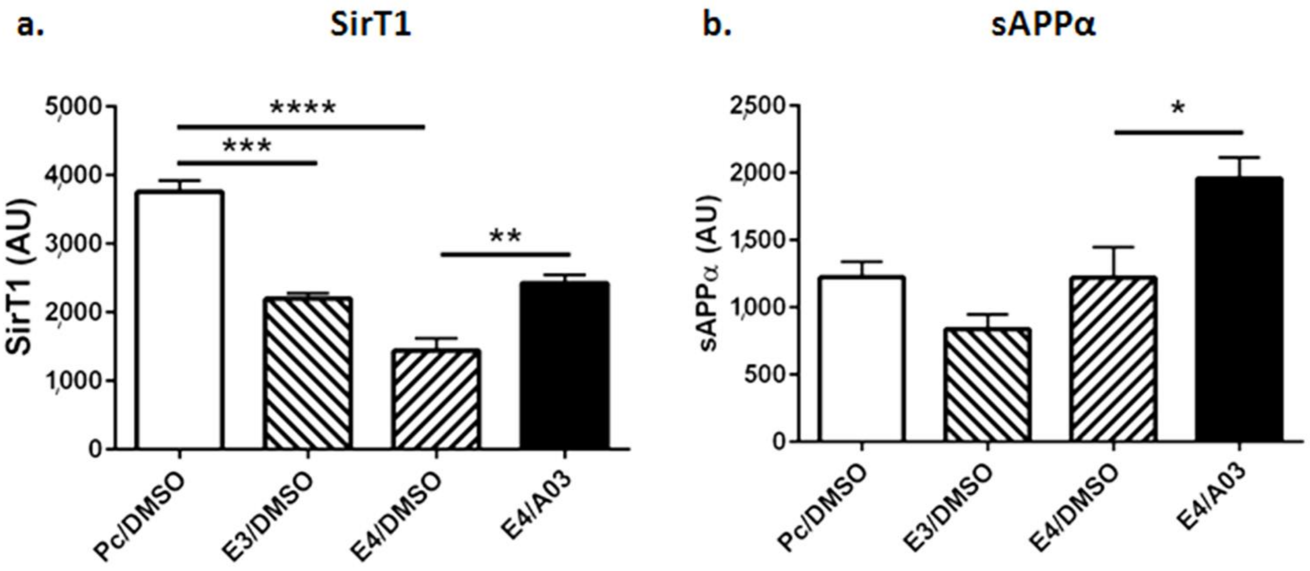

Supplementary Figure S2

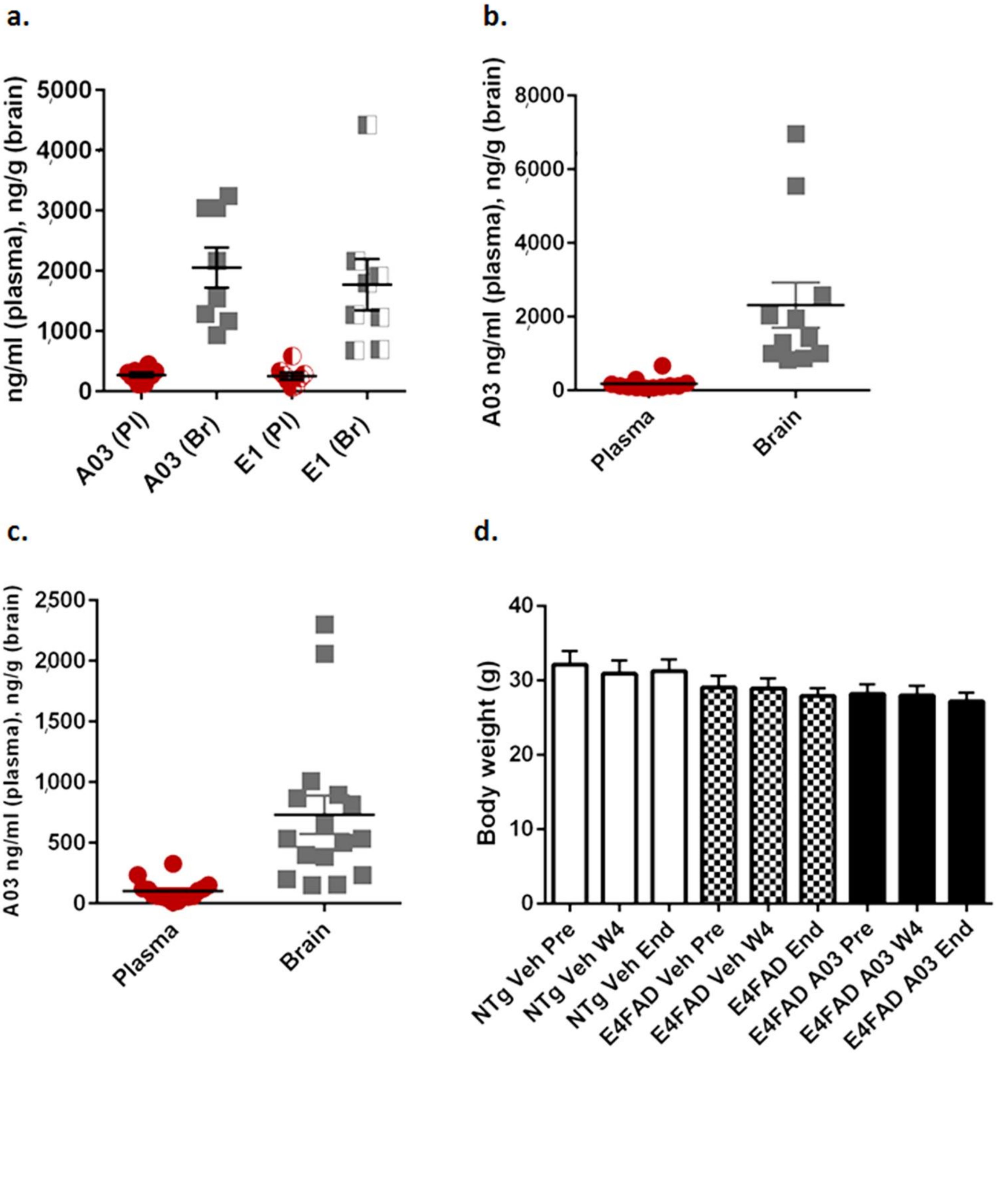

Supplementary Figure S3

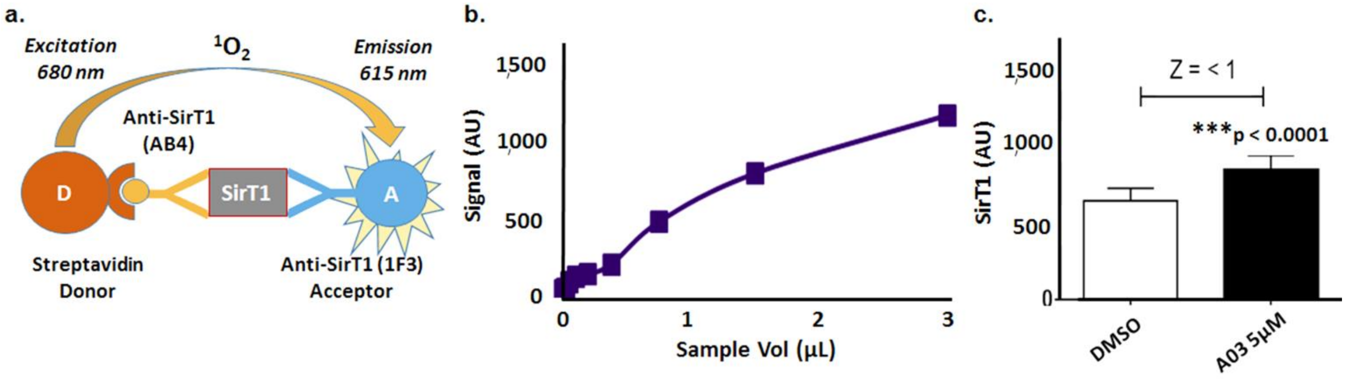

Supplementary Figure S4

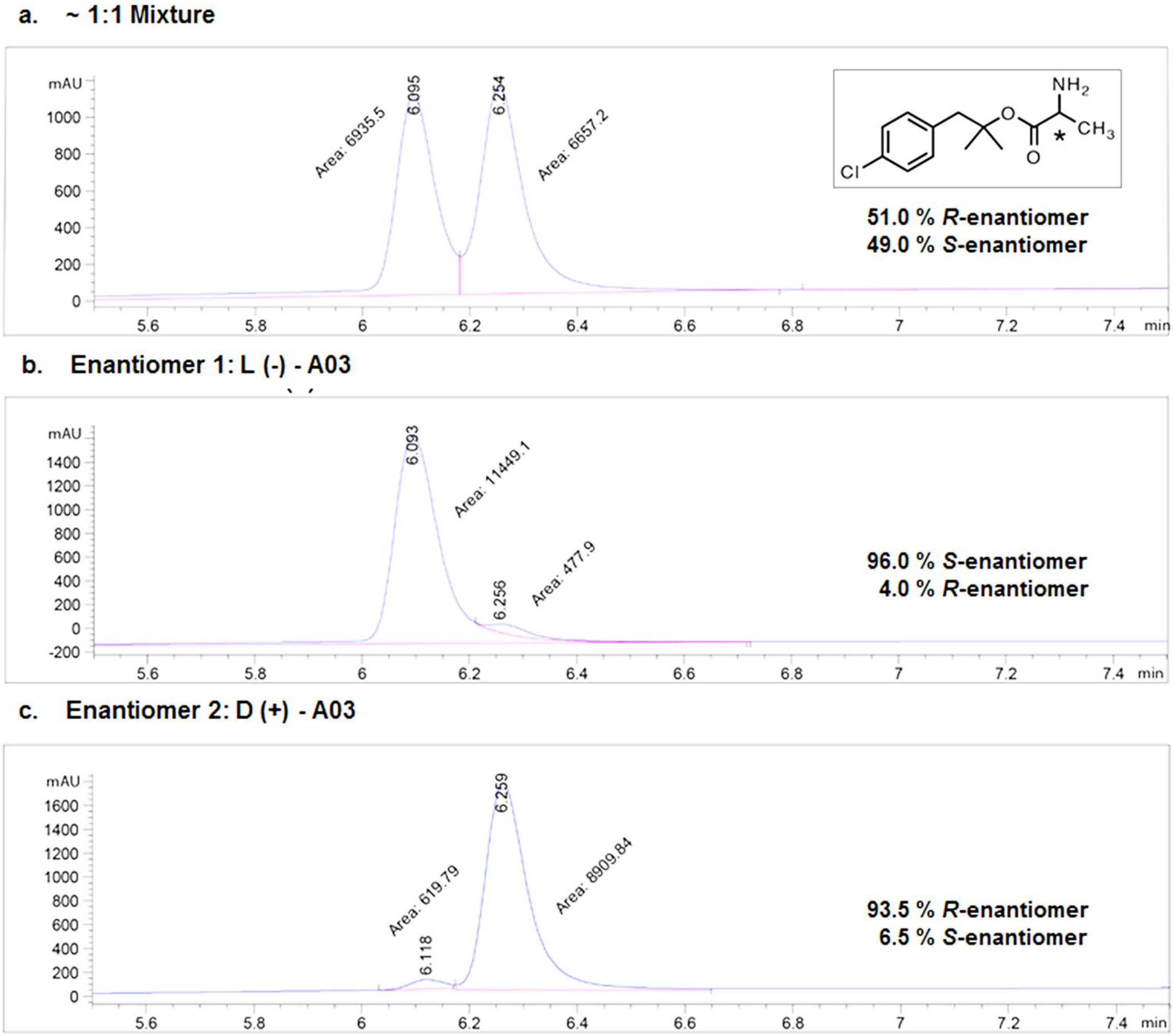

Supplement: Supplementary file 1 — Supplementary Infomation [file 41598_2018_35687_MOESM1_ESM.pdf]
